# The Proinflammatory Secretome of Senescent Cells Can Be Controlled by a HIF2A‐Dependent Upregulation and a FURIN‐Dependent Cleavage of the ANGPTL4 Secreted Factor

**DOI:** 10.1111/acel.70307

**Published:** 2025-12-05

**Authors:** Gabriela Makulyte, Hasan Safwan‐Zaiter, Delphine Goehrig, Anda Huna, Adèle Mangelinck, Takumi Mikawa, Alberta Palazzo, Lyvia Moudombi, Jean‐Jacques Medard, Marie Chanal, Pacôme Lecot, Marie‐Cécile Michallet, Julie Gavard, Serge Adnot, Pierre Dubus, Hiroshi Kondoh, Carl Mann, Nguan Soon Tan, Philippe Bertolino, Jean‐Michel Flaman, David Bernard

**Affiliations:** ^1^ Cancer Research Center of Lyon, INSERM U1052, CNRS UMR 5286, Léon Bérard Center, Lyon University Lyon France; ^2^ Equipe Labellisée la Ligue Contre le Cancer Lyon France; ^3^ Université Paris‐Saclay, CEA, CNRS, Institute for Integrative Biology of the Cell (I2BC) Paris France; ^4^ Geriatric Unit, Graduate School of Medicine Kyoto University Kyoto Japan; ^5^ Department of Diabetes, Endocrinology and Nutrition, Graduate School of Medicine Kyoto University Kyoto Japan; ^6^ Team SOAP, CRCI2NA, Nantes Université, Inserm, CNRS, Université dAngers Nantes France; ^7^ Equipe Labellisée Ligue Nationale Contre le Cancer Paris France; ^8^ Institut de Cancérologie de lOuest (ICO) Saint‐Herblain France; ^9^ Inserm U955, Département de Physiologie—Explorations Fonctionnelles, Hôpital Henri Mondor, AP‐HP, FHU SENEC Créteil France; ^10^ Institute of Lung Health Justus Liebig University Giessen Germany; ^11^ Bordeaux Institute of Oncology, BRIC U1312, INSERM, Université de Bordeaux, CHU de Bordeaux Bordeaux France; ^12^ Lee Kong Chian School of Medicine and School of Biological Sciences Nanyang Technological University Singapore Singapore Singapore

**Keywords:** age‐related diseases, ANGPTL4, cancer, cellular senescence, inflammation, senescence‐associated secretory phenotype

## Abstract

Senescent cells are characterized by a stable proliferation arrest and a senescence‐associated secretory phenotype or SASP. Although these cells can have some beneficial effects, including protecting from tumor formation, their accumulation is deleterious during aging as it promotes age‐related diseases, including cancer initiation and progression. Although the SASP has a critical role, its composition, regulation and dual role in cancer remain largely misunderstood. Here, we show that ANGPTL4 is one of the rare secreted factors induced in many different types of senescent cells. Importantly, ANGPTL4 knockdown during senescence or its constitutive expression, respectively inhibits or induces classical proinflammatory SASP factors, such as IL1A, IL6 and IL8. The latter effect is mediated upstream of IL1A, an early SASP factor, suggesting an upstream role of ANGPTL4 in SASP induction. This ANGPTL4‐dependent proinflammatory SASP can promote human neutrophil activation in ex vivo assays, or tumor initiation in a KRAS‐dependent lung tumorigenesis model in mice. This upstream activity of ANGPTL4 in regulating the proinflammatory SASP depends on its upregulation following a hypoxia‐like response and HIF2A activation, and its proteolytic processing by the FURIN proprotein convertase. Altogether these findings shed light on a two‐step activation of ANGPTL4 by HIF2A and FURIN in senescent cells and its upstream role in promoting the proinflammatory SASP, cancer and potentially other senescence‐associated diseases.

## Graphical abstract

This article supports that ANGPTL4 secreted factor is induced transcriptionally and activated by proteolytic cleavage during senescence, contributing to the production of a pro‐inflammatory SASP. In the senescent cell microenvironment, ANGPTL4 could thus contribute to senescence‐dependent alterations such as inflammation and tumor initiation.

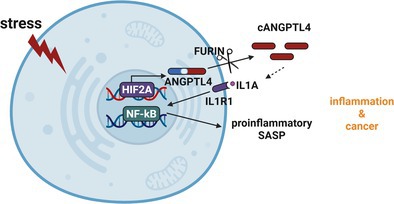

## 1 Introduction

Cellular senescence participates in the regulation of many pathophysiological responses. While senescent cells in embryos and young individuals are mostly beneficial, their accumulation during aging promotes many age‐related diseases, such as neurodegenerative diseases, cardiovascular diseases and cancers, aging being the most important risk factor for cancer overall (Alimirah et al. 2020; Gorgoulis et al. 2019; Kohli et al. 2022; McHugh and Gil 2018; He and Sharpless 2017).

Cellular senescence is induced by many signals including short telomeres, oxidative stress, DNA damaging agents or oncogene activation. Senescent cells enter a stable cell cycle arrest and display a specific secretome, named the senescence‐associated secretory phenotype or SASP. Cellular senescence acts as a fail‐safe program in order to inhibit tumorigenesis by stopping the proliferation of damaged cells and by inducing their clearance through the SASP (Collado and Serrano 2010; Collado et al. 2005). However, emerging evidence suggests that the accumulation of senescent cells, including during aging, due to decreased elimination and/or increased induction to pro‐senescent stressors, can promote long‐term cancer development and other age‐related diseases. Cancer development might be due to intrinsic effects, for instance increased DNA damage and instability, or oncogenic pathway activation, making senescent cells at risk of transformation if they are not eliminated by the immune system. They can also modify their microenvironment through their secretory phenotypes and by inducing chronic inflammation, fibrosis, cell proliferation or by inhibiting immune surveillance, favoring tumor formation and progression (He and Sharpless 2017; Acosta et al. 2013; Coppé et al. 2008; Kolodkin‐Gal et al. 2022; Azazmeh et al. 2020; Yoshimoto et al. 2013; Eggert et al. 2016).

The SASP, which encompasses growth factors, proteases, pro‐inflammatory cytokines and/or pro‐fibrotic factors, is considered to be particularly important in mediating beneficial and deleterious effects of senescence (He and Sharpless 2017; Acosta et al. 2013; Coppé et al. 2008; Kolodkin‐Gal et al. 2022; Azazmeh et al. 2020; Yoshimoto et al. 2013; Xie et al. 2023; Ovadya and Krizhanovsky 2014). Some critical regulators of the SASP, for instance the mTOR pathway, the NF‐κB and CEBPB transcription factors, and some critical components, such as IL1A proinflammatory factor, have been identified (Acosta et al. 2013, 2008; Laberge et al. 2015; Hoare et al. 2016). Nevertheless, the regulation, composition and role of the SASP are far from being fully understood.

In this study, we identified Angiopoietin Like 4 (ANGPTL4), as a unique secreted factor induced in different types (cells and inducers) of cellular senescence. We reveal (i) its function as an upstream SASP factor by acting upstream of IL1A to promote the production of proinflammatory SASP, (ii) its biological role in promoting inflammation, by activating neutrophils or by exerting pro‐tumoral effects on lung tumors, and (iii) its mechanism of upregulation and activation by HIF2A transcriptional activity and FURIN proprotein convertase activity, respectively.

## 2 Results

### 2.1 ANGPTL4 Is Upregulated in Senescent Cells

**FIGURE 1:**
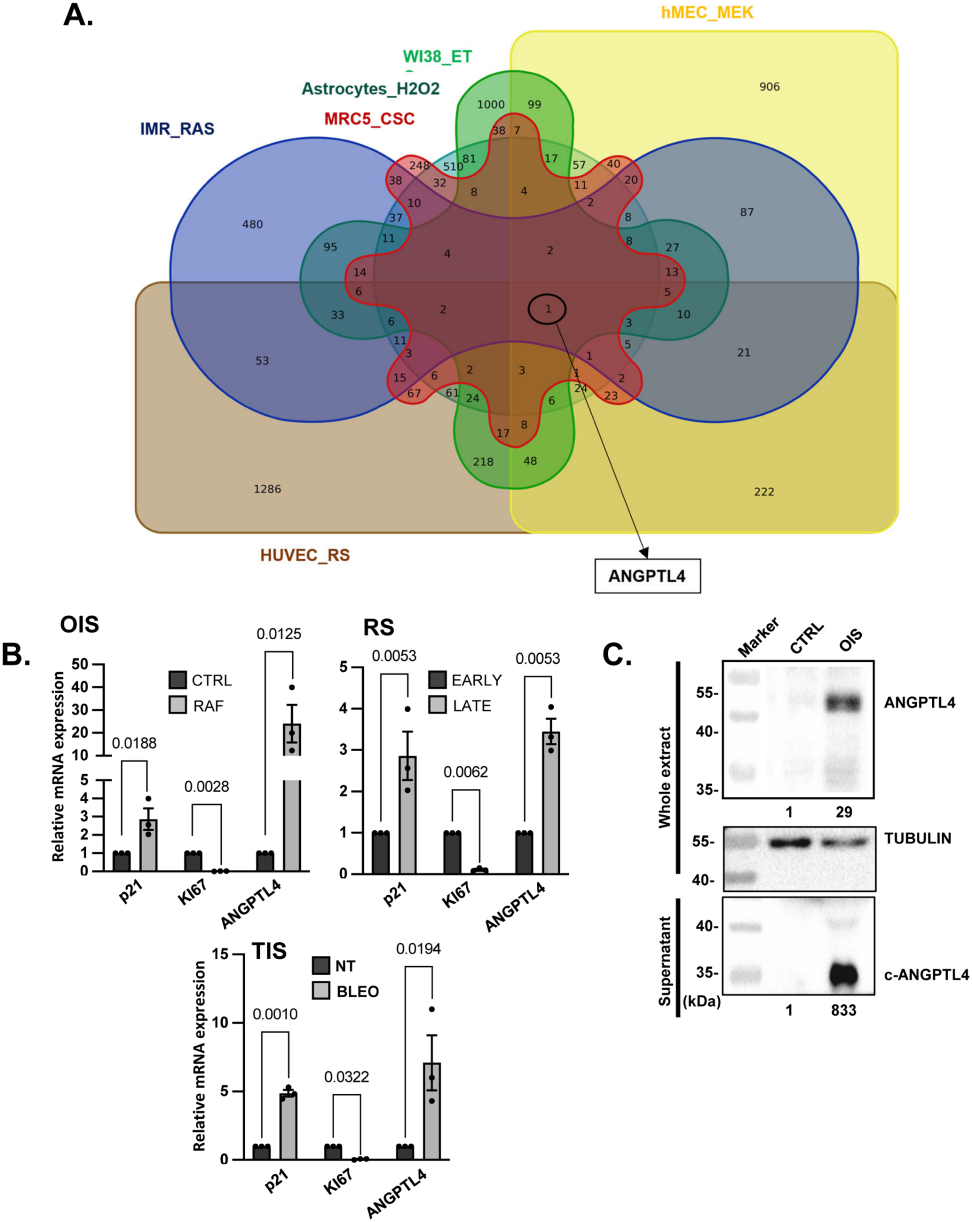
ANGPTL4 is upregulated in senescent cells. (A) Venn diagram showing genes commonly upregulated between 6 different senescence transcriptome datasets: RAS‐induced senescence in IMR90 fibroblasts (IMR_RAS); cigarette smoke condensate‐induced senescence in MRC5 fibroblasts (MRC5_CSC); etoposide‐induced senescence in WI38 fibroblasts (WI38_ET); human umbilical vein endothelial cells in replicative senescence (HUVEC_RS); MEK‐induced senescence in human mammary epithelial cells (hMEC_MEK); and H_2_O_2_‐induced senescence in astrocytes (Astrocytes_H_2_O_2_). ANGPTL4, the only gene upregulated in all conditions is indicated. (B) Relative mRNA expression of *P21*, *KI67* and *ANGPTL4* genes by using RT‐qPCR, in MRC5/RAF:ER cells untreated (CTRL) or treated with 4‐OHT (RAF) for Oncogene Induced Senescence (OIS), in p20‐p23 (EARLY) and p38‐p41 (LATE) MRC5 passages for replicative senescence (RS), and in MRC5 untreated (NT) or treated with bleomycin (BLEO) for therapy‐induced senescence (TIS). Mean ± SEM of *n* = 3 independent experiments. Paired t‐test results are indicated. (C) Western blot analysis of ANGPTL4 expression in MRC5/RAF:ER not treated (CTRL) or treated with 4‐OHT (OIS). Full‐length ANGPTL4 detected and TUBULIN loading control in whole extract and cleaved ANGPTL4 (c‐ANGPTL4) detected in cell supernatants. Representative image of *n* = 3 independent experiments.

We first sought conserved SASP factors by identifying commonly upregulated genes in 6 distinct transcriptomic datasets corresponding to different models of cellular senescence: replicative senescence of human umbilical vein endothelial cells (GSE37091 (Jong et al. 2013)), RAS‐induced senescence of IMR90 fibroblasts (GSE60652 (Takebayashi et al. 2015)), MEK‐induced senescence of human mammary epithelial cells (GSE110884 (Warnier et al. 2018)), cigarette smoke extract‐induced senescence of MRC5 fibroblasts (GSE252801 (Palazzo et al. 2023)), H_2_O_2_‐induced senescence in astrocytes (GSE58910 (Crowe et al. 2016)), and etoposide‐induced senescence in WI38 fibroblasts (GSE62701 (Contrepois et al. 2017)). Among these datasets, only 1 gene, *ANGPTL4*, showed a significant increase (> 2‐fold) in the six datasets (Figure 1A). ANGPTL4 was recently described in the SASP of hepatic stellate cells during replicative senescence (Odagiri et al. 2019), of prostate fibroblasts during DNA damage‐induced senescence (Zhang et al. 2018), of epithelial cells during centrosome amplification‐induced senescence (Wu et al. 2023), or of cancer cells during CDK4/6 inhibitor‐induced senescence (Gleason et al. 2023), confirming its induction in many cellular senescence models. Nevertheless, its conserved induction during senescence, its role in senescent cells and the associated paracrine effects, as well as its mechanism of induction in senescent cells are largely unknown. We measured ANGPTL4 mRNA levels in MRC5 normal human lung fetal fibroblasts by inducing different types of senescence: oncogene‐induced senescence (OIS) by expressing RAF oncogene, replicative senescence (RS) and therapy‐induced senescence (TIS) using bleomycin. ANGPTL4 was upregulated by all pro‐senescent signals tested (Figure 1B). These results were further confirmed as the full‐length ANGPTL4 protein was upregulated in whole cell lysates of RAF‐induced senescent cells (upper panel, Figure 1C) and the secreted cleaved C‐terminal part of ANGPTL4 was specifically induced in the supernatant of these senescent cells (lower panel, Figure 1C). Similar results were obtained in MRC5 cells during TIS induced by Etoposide (Figure S1A,B) or during OIS induced by the RAS oncogene (Figure S1C,D), and in adult human dermal fibroblasts (HDF) during OIS induced by the RAF oncogene (Figure S2A,B). These results thus demonstrate that ANGPTL4 is upregulated in different models of cellular senescence.

### 2.2 ANGPTL4 Promotes the Production of Proinflammatory SASP During Senescence

**FIGURE 2:**
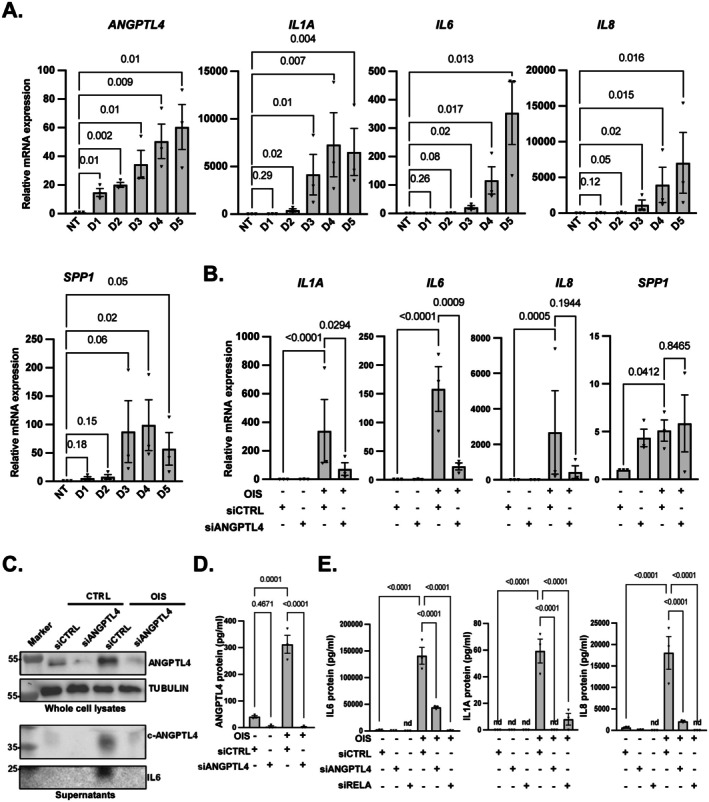
ANGPTL4 promotes production of the proinflammatory SASP during senescence. (A) RT‐qPCR analysis of ANGPTL4 and other SASP members without RAF activation (NT) or at different time points after RAF activation, from day 1 (D1) to day 5 (D5), in MRC5/RAF:ER cells. Mean ± SEM of *n* = 3 independent experiments. Paired one‐way ANOVA tests are shown. (B–D) MRC5/RAF:ER cells were transfected with control (siCTRL) or ANGPTL4 (siANGPTL4) siRNA and treated (+) or not (−) with 4‐OHT to induce senescence (OIS). (B) RT‐qPCR analysis of the gene expression of SASP members. Mean ± SEM of *n* = 3 independent experiments. Paired one‐way ANOVA tests are shown. (C) Western blot analysis of ANGPTL4 and IL6 proteins. Representative image of *n* = 3 independent experiments. (D, E) Quantification of ANGPTL4 (D) and of IL1A, IL6 and IL8 SASP factors (E) performed by ELISA. Mean protein concentrations measured in cell culture medium are ± SEM of *n* = 3 independent experiments. Paired one‐way ANOVA test results are indicated.

A temporal analysis performed after RAF activation indicated that ANGPTL4 upregulation occurred earlier than all the other tested SASP factors, namely IL1A, IL6, IL8 or SPP1 (Figure 2A), supporting that it might have a pioneer and functional role in the senescent phenotype and/or its autocrine and paracrine effects. Without oncogene activation, ANGPTL4 knockdown (Figure S3A) slightly increased senescence‐associated β‐galactosidase (SA‐β‐Gal) activity and decreased the number of cells, suggesting that its dysregulation could induce weak signs of senescence (Figure S3B–D). Strikingly, upon oncogenic stress ANGPTL4 knockdown did not impact senescence‐associated cell cycle arrest or SA‐β‐Gal activity (Figure S3B,C), but it strongly inhibited IL1A, IL6 and IL8 proinflammatory SASP components at mRNA levels (Figure 2B). These results were confirmed at the protein levels on IL6 by Western blot on cell lysates and cell culture supernatants (Figure 2C) and by ELISA on IL1A, IL6 and IL8 on supernatants (Figure 2D,E). Conversely to the proinflammatory SASP program, upregulation of the non‐proinflammatory SPP1 SASP mRNA was independent of ANGPTL4 (Figure 2B). In line with ANGPTL4 loss‐of‐function experiments, constitutive expression of ANGPTL4 induced expression of the proinflammatory SASP program as assessed by measuring IL1A and IL8 mRNA levels (Figure S3E,F). This constitutive expression also slightly increased other signs of senescence such as p21 and p16 mRNA levels, as well as SA‐β‐Gal positive cells, and decreased the number of cells (Figure S3G–I).

These results support that ANGPTL4 primarily regulates the SASP as its constitutive expression induces, and its knockdown represses, SASP production during OIS. Nevertheless, ANGPTL4 miss‐regulation (up or down) alone, without a senescent stress, only slightly increases some of the senescence marks. The early induction of ANGPTL4 seems to contribute to the proinflammatory SASP program upon oncogenic stress, which is known to be NF‐κB‐dependent (Acosta et al. 2008; Ferrand et al. 2015), and not to SPP1, a non‐proinflammatory NF‐κB‐independent SASP component (Pazolli et al. 2012).

### 2.3 ANGPTL4 Acts Upstream of IL1A to Activate the Proinflammatory SASP

**FIGURE 3:**
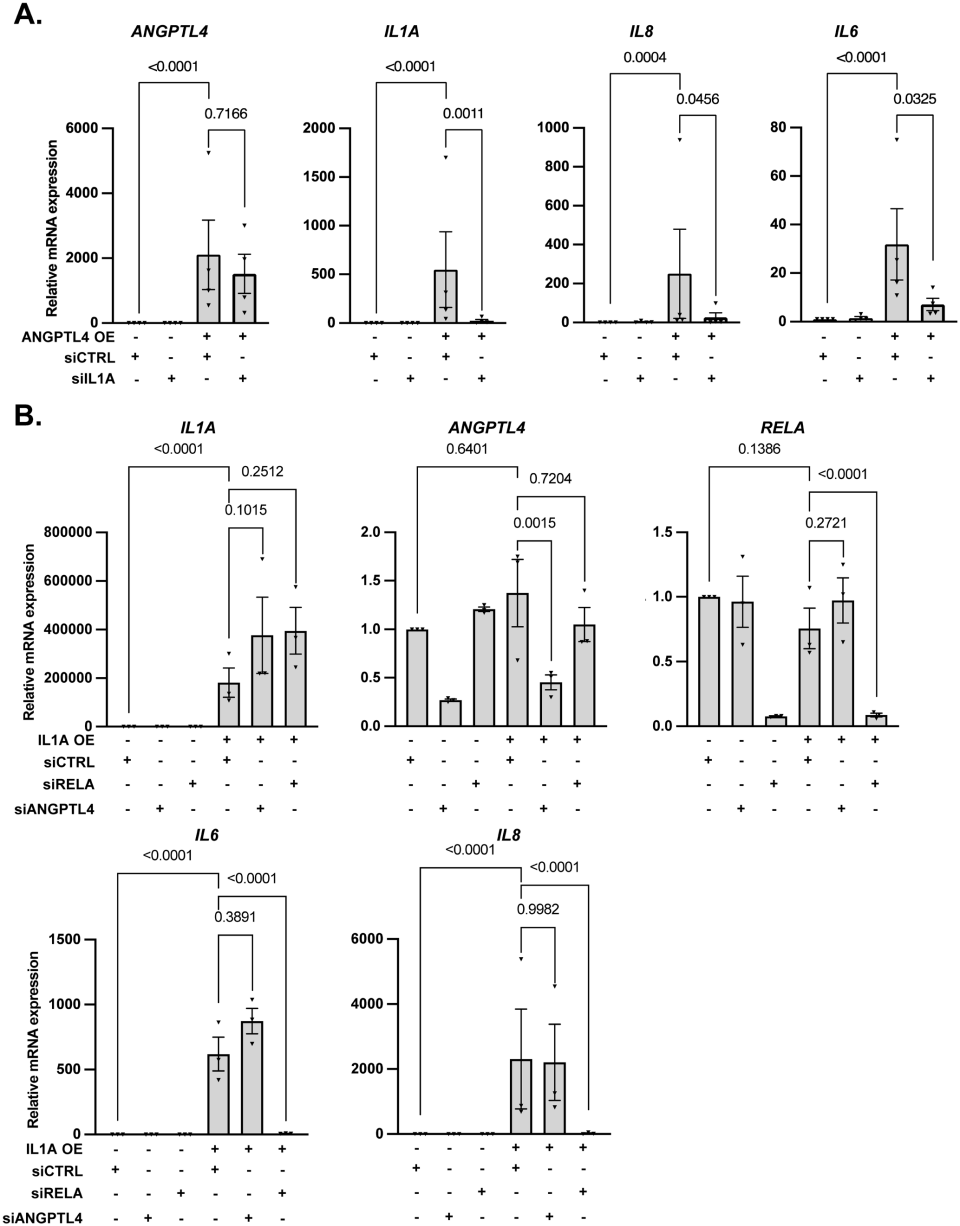
ANGPTL4 acts upstream of IL1A to activate the transcriptional proinflammatory SASP program. (A) MRC5 cells were transfected with control (siCTRL) or siRNA targeting IL1A (siIL1A) during seeding. The days after, cells were infected with control or ANGPTL4 encoding retroviral vectors. Three days later, RNAs were prepared. Relative mRNA expression of ANGPTL4 and proinflammatory SASP member genes was measured by RT‐qPCR. (B) MRC5 cells were transfected with control (siCTRL) or siRNA targeting RELA (siRELA) or ANGPTL4 (siANGPTL4) during seeding. The days after, cells were infected with control or IL1A encoding lentiviral vectors. Three days later, RNAs were prepared. Relative mRNA expression of *ANGPTL4, RELA* and proinflammatory SASP member genes was measured by RT‐qPCR Mean ± SEM of *n* = 3 independent experiments. Paired one‐way ANOVA test results are shown.

The proinflammatory part of the SASP largely depends on IL1A and subsequent activation of the NF‐κB signaling pathway for proinflammatory SASP production and amplification (Acosta et al. 2013; Laberge et al. 2015). The above results suggest that ANGPTL4 contributes to the regulation of the NF‐κB‐dependent transcriptional SASP program. To ascertain whether ANGPTL4 acts downstream or upstream of IL1A in the regulation of the proinflammatory SASP program, we tested the dependency on ANGPTL4 or IL1A following the induction of the proinflammatory SASP program by constitutively expressing IL1A or ANGPTL4, respectively. We first confirmed that IL1A upregulates the proinflammatory SASP in senescent MRC5 cells (Figure S4A). Secondly, we observed that IL1A silencing blocked the upregulation of IL6 and IL8 mRNA induced by the constitutive expression of ANGPTL4 (Figure 3A). In contrast, ANGPTL4 knockdown, unlike RELA knockdown which is used as a positive control, did not prevent the induction of the proinflammatory SASP following the constitutive expression of IL1A (Figure 3B). Lastly, the constitutive expression of ANGPTL4 led to the upregulation of IL1A mRNA (Figure 3A), whereas IL1A was unable to induce ANGPTL4 mRNA expression (Figure 3B). The results support that ANGPTL4 critically contributes to the production of the proinflammatory SASP upstream of IL1A. Nevertheless, even if the constitutive expression of ANGPTL4 and IL1A were functionally equivalent for proinflammatory SASP induction, their effects on cell proliferation were different. Indeed, whereas ANGPTL4 constitutive expression decreased proliferation (Figure S3E–I), IL1A constitutive expression did not (Figure S4B,C), supporting that ANGPTL4 can exert some effects independently of IL1A.

**FIGURE 4:**
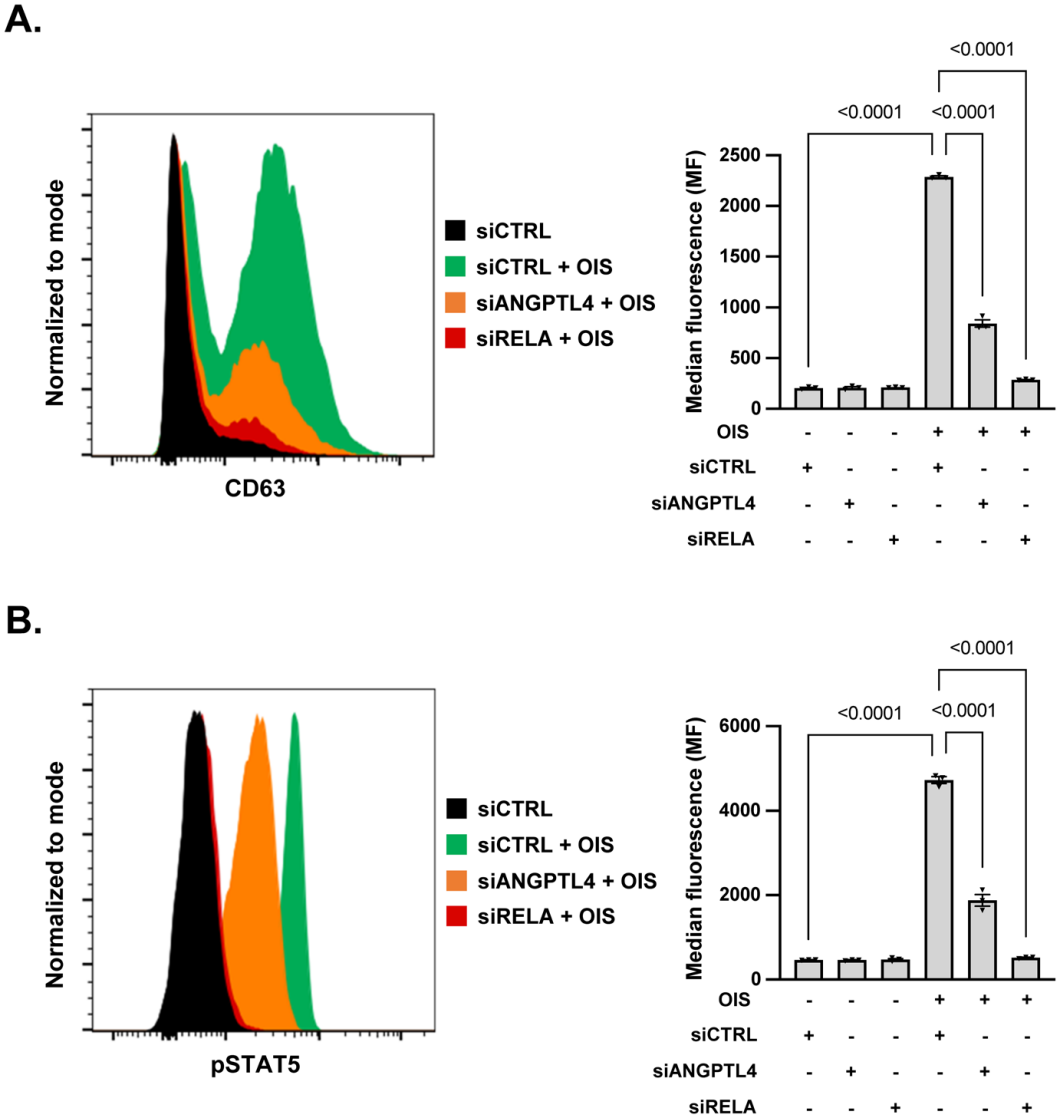
Knockdown of ANGPTL4 inhibits the ability of the SASP to induce activation of human neutrophils, a mark of inflammation. (A, B) Supernatants of MRC5/RAF:ER cells transfected with control (siCTRL), ANGPTL4 (siANGPTL4) or RELA (siRELA) siRNA and treated (+) or not (−) with 4‐OHT to induce senescence (OIS) were applied to primary human neutrophils. (A) Analysis of CD63 neutrophils degranulation marker. A representative flow cytometry profile is shown. Histograms on the right panel are the quantification of MFI. Mean ± SEM of *n* = 3 independent experiments. Paired one‐way ANOVA test results are shown. (B) Analysis of STAT5 pathway activation according to phosphorylation of STAT5. The left panel displays a representative flow cytometry profile. Histograms on the right panel are the quantification of MFI (Mean Fluorescence Intensity). Mean ± SEM of *n* = 3 independent experiments. Paired one‐way ANOVA test results are shown.

As we previously showed that the SASP can activate neutrophils (Debiesse et al. 2025) and that they are key regulators of inflammation (Rawat and Shrivastava 2022), we next used an ex vivo assay to assess the ability of the ANGPTL4‐dependent secretome of senescent cells to activate inflammation or not by measuring primary human neutrophil activation. Secretomes were produced from MRC5 control and senescent cells with or without a knockdown of ANGPTL4 or RELA, used as a positive control to inhibit the proinflammatory SASP (Figure 2E and Figure S5). As expected, the SASP was able to induce features of inflammation as assessed by increased CD63 (Figure 4A) and phosphorylation of the STAT5 signaling molecule (Figure 4B), both being hallmarks of activated neutrophils (Futosi et al. 2013; Lopez et al. 1995). Strikingly, ANGPTL4 knockdown, as for RELA knockdown, largely reverted the ability of the secretome of senescent cells to activate neutrophils (Figure 4A,B). These results support that the paracrine proinflammatory effect of the secretome of senescent cells involves the ANGPTL4 factor.

### 2.4 A Hypoxia‐Like Response Mediated by HIF2A Upregulates ANGPTL4 During Senescence

We then tested the involvement of NF‐κB and CEBPB transcription factors, well‐known regulators of the expression of multiple SASP factors (Acosta et al. 2008; Ferrand et al. 2015), in the regulation of ANGPTL4 during senescence. Although the knockdown of RELA, a key NF‐κB subunit, or of CEBPB strongly decreased the expression of IL1A, IL6 and IL8 mRNA during OIS, they did not impact ANGPTL4 mRNA upregulation (Figure S6), ruling out their involvement in the regulation of ANGPTL4 during OIS and suggesting alternative mechanisms of ANGPTL4 regulation.

**FIGURE 5:**
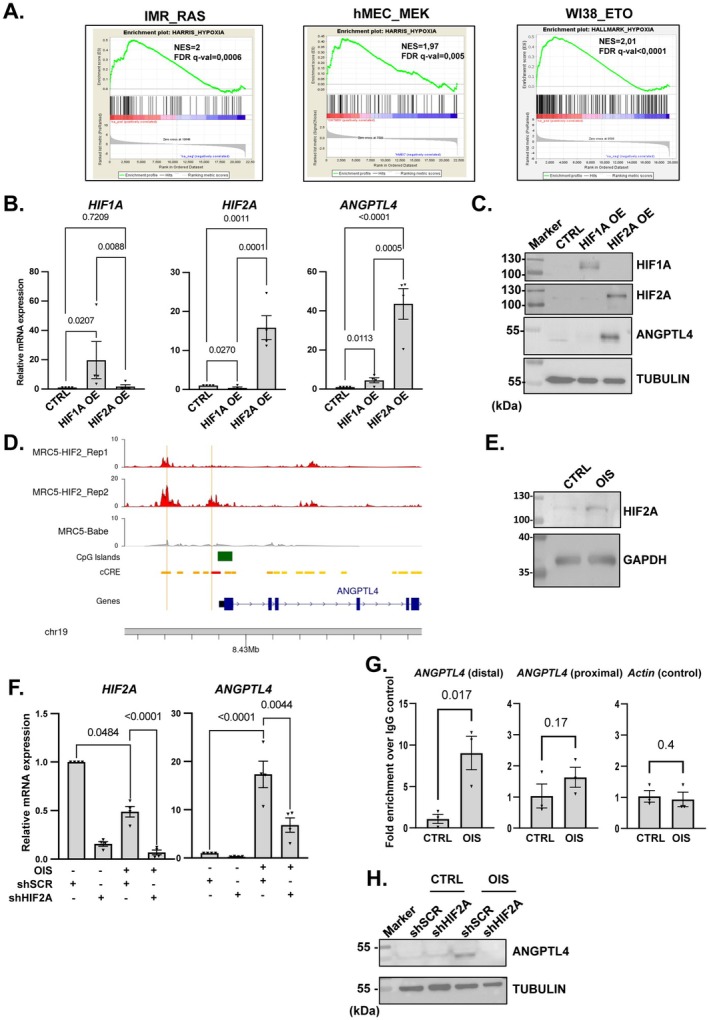
A hypoxia‐like response mediated by HIF2A upregulates ANGPTL4 during senescence. (A) GSEA plots showing enrichment of the HYPOXIA gene set in 3 senescence models: RAS‐induced senescence in IMR90 (IMR90‐RAS) MEK‐induced senescence in human mammary epithelial cells (hMEC_MEK), and etoposide‐induced senescence in WI38 (WI38‐ETO). Normalized Enrichment Score (NES) and FDR q‐value are indicated. (B, C) MRC5 cells were infected with an empty vector (CTRL), or HIF1A (HIF1A OE) and HIF2A (HIF2A OE) expressing vectors. (B) Relative mRNA expression of *HIF1A*, *HIF2A* and *ANGPTL4* genes by RT‐qPCR. Mean ± SEM of *n* = 4 independent experiments. Paired one‐way ANOVA values are indicated. (C) Western blot analysis of HIF1A, HIF2A, ANGPTL4 and the loading control TUBULIN. Representative picture of *n* = 3 independent experiments. (D) HIF2A peaks identified by Cut & Tag at the *ANGPTL4* promoter in MRC5 cells infected with HIF2A (HIF2) or control (Babe) retroviral particles. Two independent experiments were performed (HIF2A‐Rep1, HIF2A‐Rep2). Both peaks contain a Hypoxia Response Element (ACGTG). (E) Western blot analysis of ANGPTL4 and the loading control GAPDH during RAF‐induced senescence (OIS). Representative picture of *n* = 3 independent experiments. (F) MRC5/RAF:ER cells were infected with lentiviral vectors encoding scramble (shSCR) or HIF2A shRNA (shHIF2A) and next treated (+) or not (−) with 4‐OHT to induce senescence (OIS). Relative mRNA expression of *HIF2A* and *ANGPTL4* genes by RT‐qPCR. Mean ± SEM of *n* = 4 independent experiments. Paired one‐way ANOVA test results are shown. (G) ChIP‐qPCR assay to assess endogenous HIF2A binding on the *ANGPTL4* promoter during OIS induced by RAF. Chromatin fractions derived from 4‐OHT‐treated and untreated MRC5/RAF:ER cells were subjected to immunoprecipitation with anti‐HIF2A antibody or IgG control. Primer sets were designed for regions 2179 bp (distal) and 369 bp (proximal) upstream of the *ANGPTL4* TSS, and for *Actin* promoter as a control. Mean ± SEM of *n* = 3 independent experiments. Paired *t*‐test values are indicated. (H) Western blot analysis of ANGPTL4 and the loading control TUBULIN. Representative picture of *n* = 3 independent experiments.

ANGPTL4 was previously reported to be upregulated in cancer cell lines by the HIF1A (Hypoxia Inducible Factor 1 Subunit Alpha) transcription factor during hypoxia (Baba et al. 2017; Li et al. 2011). Strikingly, Gene Set Enrichment Analysis (GSEA) of transcriptomic datasets from senescent cells revealed a significant enrichment of genes involved in the hypoxia signaling pathway during OIS (Figure 5A). As HIF1A and HIF2A transcription factors are the main drivers of the hypoxic response, we tested the ability of their constitutive expression to induce ANGPTL4 expression in MRC5 fibroblasts. Only the constitutive expression of HIF2A strongly induced ANGPTL4 expression at mRNA (Figure 5B) and protein (Figure 5C) levels. Cut & Tag analysis (Kaya‐Okur et al. 2020) on HIF2A in MRC5 fibroblasts constitutively expressing HIF2A showed binding of HIF2A mainly around the TSS of promoters (Figure S7A,B). Notably, we observed binding of HIF2A to promoter regions of the *ANGPTL4* promoter (Figure 5D) and the *VEGFA* promoter (Figure S7C), a classical target of HIF proteins, at regions containing Hypoxia Response Elements, but not to the promoters of proinflammatory SASP genes (Figure S7D). The HIF2A protein was slightly increased during OIS (Figure 5E) whereas its mRNA level displayed a slight decrease (Figure 5F). Using primers targeting the HIF2A binding site identified by the Cut & Tag analysis, we performed ChIP‐qPCR analysis and observed an increased binding of endogenous HIF2A on the *ANGPTL4* promoter during OIS (Figure 5G). Consistently, HIF2A knockdown significantly reverted ANGPTL4 upregulation during RAF‐induced senescence in MRC5 cells at mRNA and protein levels (Figure 5F,H), whereas HIF1A knockdown did not impact ANGPTL4 mRNA levels (Figure S7E).

Hence, these results support a critical role for the HIF2A transcription factor in ANGPTL4 upregulation during senescence.

### 2.5 Proteolytic Cleavage of ANGPTL4 by FURIN Is Required for Activation of the SASP

**FIGURE 6:**
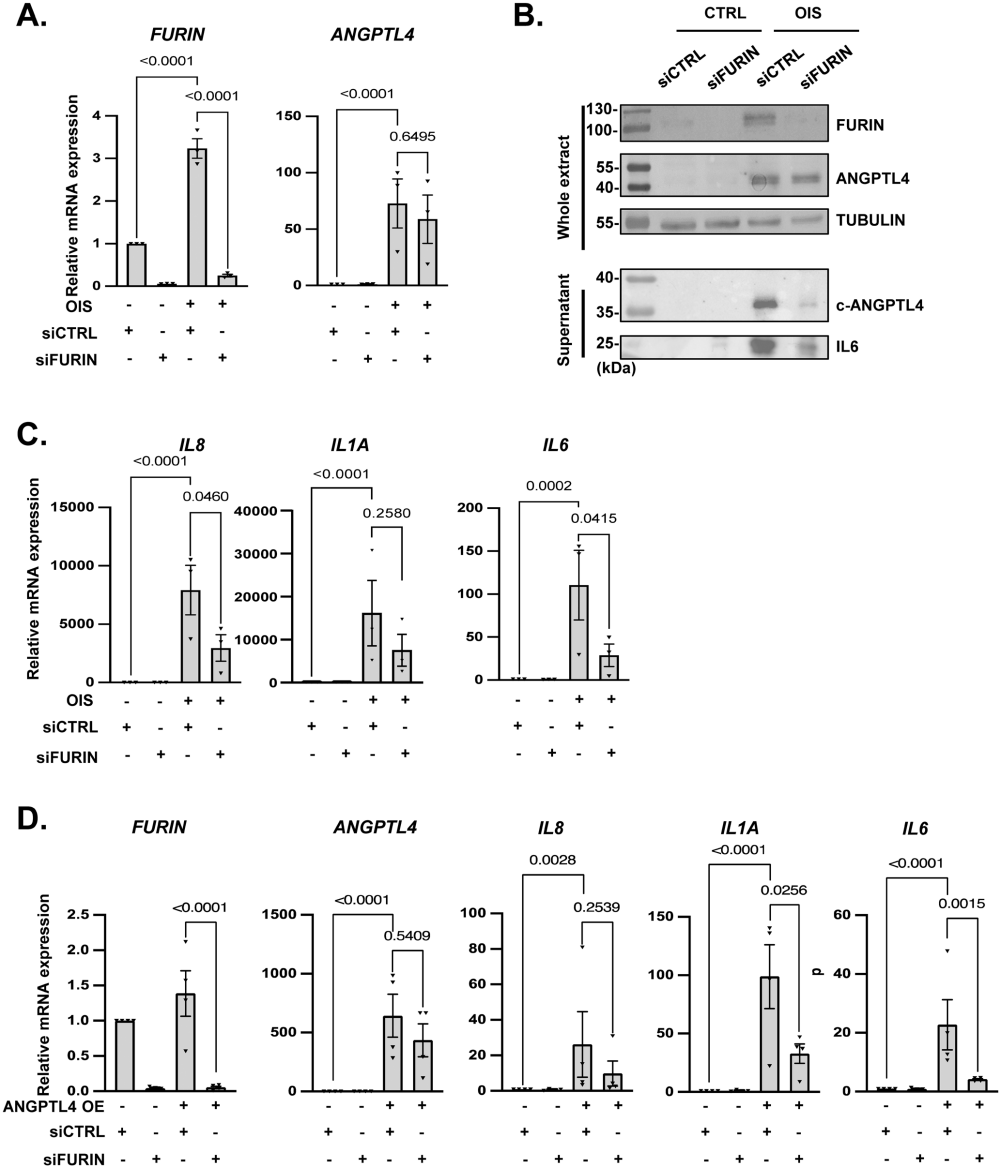
Cleavage of ANGPTL4 by FURIN is required for its ability to promote the proinflammatory SASP. (A‐C) MRC5/RAF:ER cells were transfected with control (siCTRL) or FURIN (siFURIN) siRNA and treated (+) or not (−) with 4‐OHT to induce senescence (OIS). (A) RT‐qPCR analysis against the indicated genes 3 days after 4‐OHT treatment. Mean ± SEM of *n* = 3 independent experiments. Paired one‐way ANOVA test results are shown. (B) Western blot analysis of FURIN, ANGPTL4: Full length in whole cell extracts (ANGPTL4) and secreted in the supernatant (cANGPTL4) and IL6. Representative picture of *n* = 3 independent experiments. (C) RT‐qPCR analysis of the indicated SASP encoding genes. Mean ± SEM of *n* = 3 independent experiments. Paired one‐way ANOVA test values are shown. (D) Relative mRNA expression of *FURIN*, *ANGPTL4* and proinflammatory SASP encoding genes measured by RT‐qPCR in MRC5 cells overexpressing or not ANGPTL4 (ANGPTL4 OE) and transfected with control (siCTRL) or FURIN (siFURIN) siRNA. Mean ± SEM of *n* = 4 independent experiments. Paired one‐way ANOVA test results are shown.

Given the role of several PCSK (Proprotein Convertase Subtilisin/Kexin) proteins in ANGPTL4 activation (Lei et al. 2011), and that cleaved ANGPTL4 was observed in the SASP (Figure 1C), along with another PCSK substrate, INHBA (Figure S8A) (Antenos et al. 2011; Pinjusic et al. 2024), we wondered whether this proteolytic activation is required for the induction of the proinflammatory SASP during senescence or by constitutive expression of ANGPTL4. We analyzed the expression of the PCSKs after RAF activation and observed the induction of FURIN and PCSK5 messengers shortly after RAF activation according to our transcriptomic dataset (Figure S8B). Furin RNAs upregulation, but not PCSK5 one, was also observed after RAF activation at a later time point (Figure 6A and Figure S8C). To assess whether these proteins cleaved ANGPTL4 upon OIS, we knocked down FURIN and PCSK5 proprotein convertase, which had no impact on ANGPTL4 mRNA levels, but FURIN knockdown strongly impaired c‐ANGPTL4 and IL6 levels in the secretome of senescent cells (Figure 6B and Figure S8D), whereas PCSK5 knockdown had a weak impact (Figure S8D). We then focused on FURIN and observed that its knockdown also impaired the increase in the proinflammatory SASP program in senescent cells (Figure 6C). Moreover, the induction of the proinflammatory SASP program by the constitutive expression of ANGPTL4 was largely prevented by FURIN knockdown, further supporting the involvement of ANGPTL4 proteolytic cleavage by FURIN in the regulation of the proinflammatory SASP (Figure 6D).

These data demonstrate that ANGPTL4 needs to be cleaved by FURIN to promote the proinflammatory SASP.

### 2.6 ANGPTL4 Promotes Tumor Initiation

The SASP can promote long‐term tumor initiation and progression (Acosta et al. 2013; Coppé et al. 2008; Kolodkin‐Gal et al. 2022; Azazmeh et al. 2020; Yoshimoto et al. 2013). Before experimentally investigating the involvement of ANGPTL4 in this context, we ascertained in which type of cancer it may play a role. To this end, we analyzed its expression level in a large collection of human tumors using The Cancer Genome Atlas (TCGA) datasets. Notably, non‐small cell lung cancer (NSCLC) ranked second in terms of ANGPTL4 expression levels (Figure S9A) and it is among the well‐known age‐dependent cancers. In addition, ANGPTL4 expression was higher in lung adenocarcinoma (LUAD) samples, a subtype of NSCLC, than in normal lung counterparts (Figure S9B). Its expression also increased with LUAD grade (Figure S9C) and was correlated with an increased risk of progression of the disease and patient mortality (Figure S9D). These findings suggest an important role for ANGPTL4 in lung tumors, which we then focused on.

**FIGURE 7:**
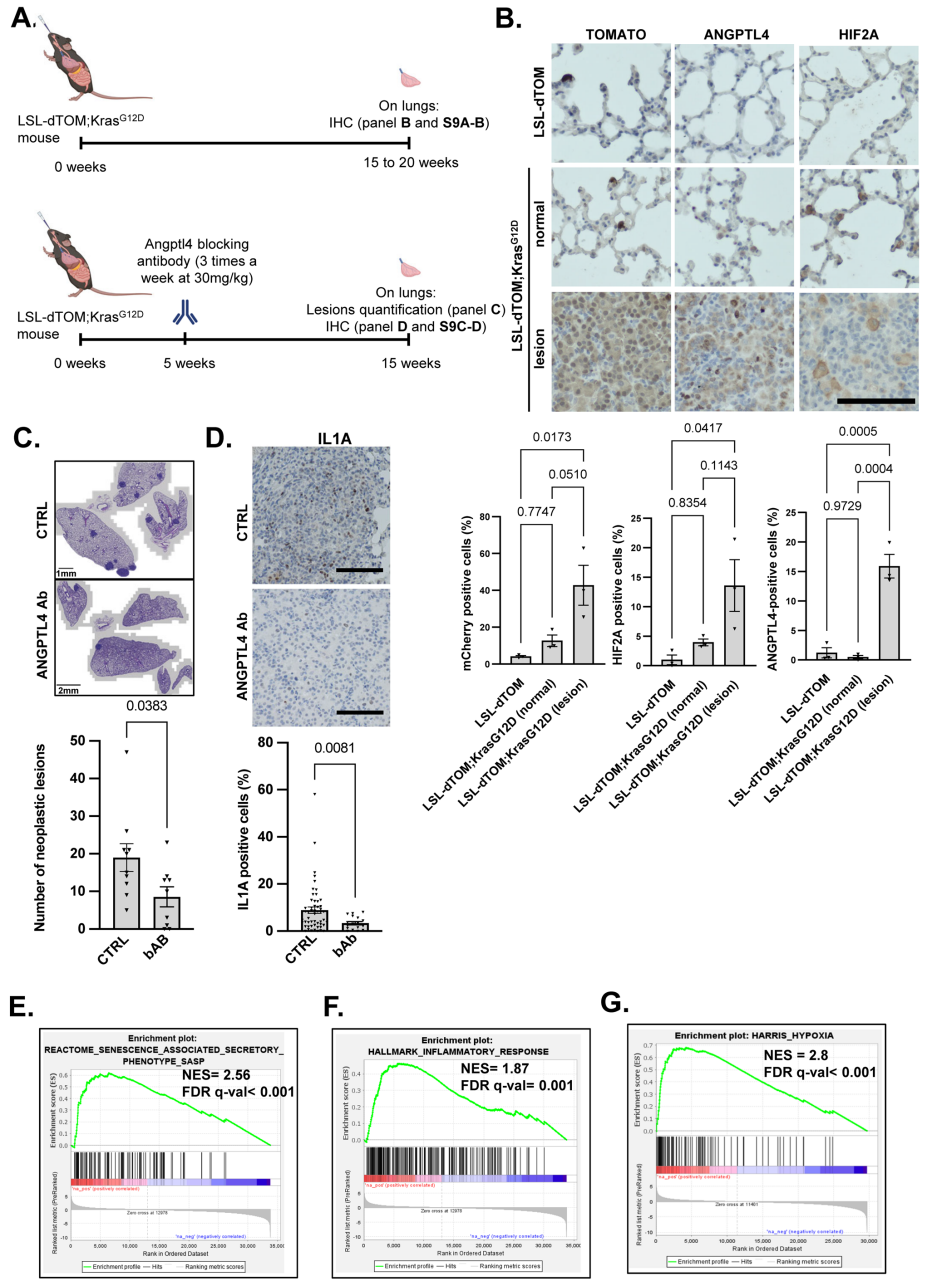
ANGPTL4 promotes proinflammatory SASP and tumorigenesis in the lung. (A) Schematic representation of the experimental design of (B–D). LSL‐dTOM;Kras^G12D^ mice were infected with a CRE‐encoding lentivirus. Lungs were retrieved and processed 15 to 20 weeks after (B), or 5 weeks post‐infection mice were treated with ANGPTL4 blocking antibody (bAb) for 10 weeks before analyses (C–D). (B) Lungs were prepared 15–20 weeks after CRE‐encoding lentivirus infection. Immunohistochemistry was performed against Tomato (to stain KrasG12D‐positive cells), ANGPTL4 and HIF2A. Representative picture of at least 10 lesions in 4 KT mice and 3 control mice. Scale bar: 100 μm. Quantifications of positive cells from 3 independent lungs are shown. (C) Neoplastic lesion quantification in mice treated with ANGPTL4 bAb (bAb) (*n* = 9) or not treated (CTRL) (*n* = 10). Left panel: Representative picture of hematoxylin–eosin staining; right panel: Quantification of neoplastic lesions. Mean ± SEM, unpaired *t*‐test. (D) Immunohistochemistry analysis of IL1A staining in mouse neoplastic lung lesions treated with ANGPTL4 bAb (bAb), *n* = 17 lesions; and not treated (CTRL), *n* = 53 lesions. Left panel: Representative picture of the staining; scale bar: 100 μm. Right panel: Quantification result, percentage of positive cells per lesion. Mean ± SEM, Mann Whitney test. (E–G) GSEA plots showing enrichment of the “REACTOME_SENESCENCE_ASSOCIATED_PHENOTYPE_SASP”, “HALLMARK_INFLAMMATORY_RESPONSE” and “HARRIS_HYPOXIA” gene sets in LUAD tumors with high ANGPTL4 mRNA expression versus low ANGPTL4 mRNA expression.

We investigated the role of ANGPTL4 in the onset of KrasG12D‐induced lung tumor using a well‐established mouse model in which the KrasG12D‐activated mutation drives tumorigenesis associated with the accumulation of senescent cells according to SA‐β‐Gal activity or expression of p21 and γH2AX (Figure 7A, upper timeline and Figure S10A,B) (Collado et al. 2005). As expected, ANGPTL4 expression increased in lung neoplastic lesions after instillation of a lentivirus encoding the CRE recombinase in LSL‐dTOM;Kras^G12D^ (KT) mice (Figure 7B). Importantly, this increase in ANGPTL4 was correlated with an increase in HIF2A, supporting that the latter could participate in the upregulation of ANGPTL4 observed in KrasG12D activated areas (Figure 7B), in line with our in vitro results. FURIN staining seemed ubiquitous and not upregulated in the lesions (Figure S10C). Next, we injected a validated blocking antibody (Li et al. 2015, 2019; Kolb et al. 2019) in KT transduced mice and assessed its impact on neoplastic lesions (Figure 7A, lower timeline). The number of neoplastic lesions significantly decreased following the administration of the ANGPTL4 blocking antibody (Figure 7C) and this was correlated with decreased proinflammatory SASP IL1A staining (Figure 7D) and CCl3 staining (Figure S10D), in line with our observations in cultured cells. Strikingly, according to GSEA analysis of the TCGA database, lung tumors with high levels of ANGPTL4 displayed significant enrichment of SASP genes (Figure 7E) and of genes involved in the inflammatory response (Figure 7F), supporting a role of ANGPTL4 in the regulation of the proinflammatory SASP in lung tumors, as observed after ANGPTL4 gain‐ or loss‐of‐function experiments. In addition, ANGPTL4 expression was also associated with a hypoxia gene set in these human lung tumors (Figure 7G), suggesting its upregulation by a hypoxia‐like pathway, as in our cells after oncogenic activation. Together these results suggest that ANGPTL4 SASP factor promotes tumor initiation.

## 3 Discussion

The secretome of senescent cells contributes to various age‐related disorders, including tumor formation and progression. Therefore, understanding the composition, function and regulation of SASP components is of paramount importance. In this study, we identified ANGPTL4 as a widely and early secreted SASP member. ANGPTL4 promotes the proinflammatory SASP program upstream of the IL1A signaling pathway suggesting its pioneer role in the SASP. Strikingly, ANGPTL4 activity in senescent cells required a novel two‐step induction: its transcriptional upregulation by HIF2A and its proteolytic cleavage by FURIN. Moreover, ANGPTL4 promoted the proinflammatory paracrine effects of the SASP by three criteria: (i) its knockdown decreases human neutrophil activation in an original ex vivo system we developed, (ii) blocking ANGPTL4 in mice decreased oncogenic Kras‐induced lung tumors, alongside a decrease in IL1A expression, and (iii) human lung tumors expressing high levels of ANGPTL4 displayed enrichment of SASP and proinflammatory gene signatures.

While ANGPTL4 upregulation in senescent cells has recently been reported in only a few studies, its functions and mechanism of regulation in senescent cells remain largely unknown (Odagiri et al. 2019; Zhang et al. 2018; Wu et al. 2023). By crossing several transcriptomic datasets of senescent cells, from different cell types and senescence inducers, we have extended those initial discoveries and emphasized that *ANGPTL4* is among a very limited number of genes significantly upregulated in different cellular senescence contexts. Besides its unique broad and potentially universal induction in senescent cells as shown in our study, ANGPTL4 participates in the regulation of pathophysiological processes that could be linked with cellular senescence, including cancer (Yan et al. 2021), fibrosis (Saito et al. 2023), inflammation (Wu et al. 2016), and infection (Li et al. 2015), reinforcing the potential significance of this SASP factor in contributing to senescence‐dependent effects.

ANGPTL4 is induced in senescent cells raising the question of its regulation in senescent cells. Contrary to the classical proinflammatory SASP components, our results indicate that ANGPTL4 upregulation is independent of NF‐κB or of CEBPB transcription factors. Hypoxia, in particular through the HIF1A transcription factor, was implicated in the expression of ANGPTL4 in cancer cells, in presumably non‐senescence contexts (Li et al. 2011; Kubo et al. 2016). A recent study also described ANGPTL4 induction by HIF1A during centrosome amplification‐induced senescence in immortal cells (Wu et al. 2023) and we show that the hypoxia gene signature is induced in other senescent contexts. However, the specific contribution of hypoxia to senescence remains under debate (Welford and Giaccia 2011; Salminen et al. 2016; Gao et al. 2023). Importantly, we find that HIF2A, rather than HIF1A, contributed to ANGPTL4. Thus, we suggest that a hypoxia‐like response is activated in senescent cells and that this response contributes to ANGPTL4 upregulation, but the involvement of HIF1A, HIF2A, or both might depend on the cell type, its transformed status and/or the senescence inducer.

The cleaved form of ANGPTL4, and not its full length, is secreted by senescent cells, so an additional level of ANGPTL4 regulation through its proteolysis might occur in senescent cells. Indeed, FURIN proprotein convertase, described to cleave ANGPTL4 in a specific context (Lei et al. 2011), participates in ANGPTL4 proteolytic processing in senescent cells, and its decrease in senescent cells or upon ANGPTL4 constitutive expression largely represses proinflammatory SASP induction. These results support that FURIN inhibition could also be a novel strategy to inhibit the proinflammatory SASP.

ANGPTL4 knockdown during OIS or its constitutive expression allowed us to identify its role in promoting the proinflammatory SASP. Indeed, it participates in the induction of IL1A, IL6 and IL8 proinflammatory SASP and not of the non‐proinflammatory SPP1 mRNA. Moreover, ANGPTL4 upregulation is not involved in proliferation arrest during senescence. Nevertheless, Koff and colleagues recently reported that ANGPTL4 contributes to the resistance of liposarcoma cells to CDK4/6 inhibitors, potentially by mitigating senescence features. Indeed, knockdown of ANGPTL4 decreases some SASP factors and the proliferation arrest induced by this treatment (Gleason et al. 2023). As the SASP can participate in proliferation arrest (Acosta et al. 2008; Kuilman et al. 2008), it is possible that the contribution of ANGPTL4 to this mechanism is mediated by its effect on the SASP during therapy‐induced senescence in cancer cells. The ability of ANGPTL4 to promote specific SASP factors without contributing to the cell cycle arrest during senescence, as we observed, is reminiscent of the effects of IL1A (Lau et al. 2019). IL1A, as ANGPTL4, is also known to regulate the proinflammatory SASP (Laberge et al. 2015; Lau et al. 2019), suggesting a potential functional link between ANGPTL4 and IL1A. Confirming a functional link between these two SASP factors, knockdown of IL1A blocks proinflammatory SASP induction upon ANGPTL4 constitutive expression. In contrast the knockdown of ANGPTL4 had no impact on the proinflammatory SASP induced by IL1A constitutive expression. Therefore, ANGPTL4 acts upstream of IL1A, a known early SASP factor (Acosta et al. 2013; Laberge et al. 2015), to regulate the proinflammatory branch of the SASP suggesting it can act as an early SASP factor. We can speculate that ANGPTL4 is acting through interaction, directly or indirectly, with a receptor. According to the Cellinker database ANGPTL4 could interact at the membrane with CDH5, CDH11, SDC1‐4, ITGA5/ITGB1, or ITGAV/ITGB3. Nevertheless, whether one of these potential ANGPTL4 receptors or other mechanisms are involved in the activation of IL1A effects will have to be determined in the future.

Senescent cells have broad effects through their proinflammatory SASP. Our results highlight the key role of ANGPTL4 in potentially regulating the inflammatory‐dependent processes. First, supernatant derived from senescent cells in which ANGPTL4 has been decreased strongly repressed the ability of this secretome to induce primary human neutrophil activation ex vivo. Second, inhibiting ANGPTL4 in vivo decreased IL1A production and tumor initiation, the two probably being functionally linked as inhibiting IL1A slows down RAS‐dependent tumor initiation in a pancreatic model (Lau et al. 2019). Last, ANGPTL4 levels correlate with SASP and inflammation gene set signatures in lung tumors. In the context of lung tumors our findings are particularly interesting owing to the accumulation of senescent cells. Indeed, cigarette smoke exposure is the main risk factor for lung tumors (Hecht 2012; Izzotti and Pulliero 2015) and favors the accumulation of senescent cells in the lung (Nyunoya et al. 2006; Sorrentino et al. 2014). We can then speculate that in smokers, the accumulation of lung senescent cells could promote long‐term lung tumor formation at least in part through ANGPTL4 induction. Additional approaches will be required to confirm the functional link between ANGPTL4 and IL1A in tumor initiation such as by assessing whether an IL1A blocking Ab phenocopies the effect of ANGPTL4 blocking Ab in the lung cancer model, and whether increased ANGPTL4 expression impacts the number of lesions, and if so its potential reversal using IL1A blocking antibody. Evaluation of the contribution of some immune cells, such as neutrophils (Debiesse et al. 2025), which can be regulated by ANGPTL4 and senescent cells and impact tumor initiation, will also be required.

Overall, our findings support that ANGPTL4 is an early and unique SASP factor by its universality, pioneer activity to promote the proinflammatory SASP and its two‐step regulation: transcription by HIF2A and proteolytic processing by FURIN, in senescent cells. Given that the regulation of the SASP has attracted much interest over the last decade to improve various age‐related diseases, including cancer (Zhang et al. 2023; Soto‐Gamez and Demaria 2017), our work paves the way for the development of preventive and therapeutic strategies blocking the deleterious effects of the proinflammatory SASP by targeting ANGPTL4 or its upstream regulators.

## 4 Materials and Methods

### 4.1 Cell Culture and Reagents

MRC5 normal human fibroblasts (ATCC), 293GP (Clonotech) and 293 T (Clontech) were cultured in DMEM (Dubelcco's Modified Eagle's Medium, Life Technologies) supplemented with 10% Fetal Calf Serum (FCS, Life Technologies) and 1% antibiotics (Penicillin, Streptomycin, Gibco). Human dermal fibroblasts (Genomic and Genetic Disorders Biobank, Italy) were maintained in DMEM/F‐12 (1:1) medium supplemented with GlutaMAX (Gibco, Cat# 31331–028), 10% fetal bovine serum (FBS; Gibco, Cat# 10437‐028), and 1% penicillin–streptomycin (Gibco, Cat# 15140‐122). The 293GP and 293 T were cultured on Poly‐L‐Lysine (Corning) coated plates, at 3 μg/mL. Cells were cultured at 37°C and 5% CO_2_. They were checked regularly for mycoplasma.

4‐hydroxytamoxifen (4‐OHT) (Sigma Aldrich) was used between 1, 10 nM for RAF and 50 nM for RAS. Bleomycin was added twice every 2 days at 3 μg/mL. Etoposide was added every 2 days at 10 μM.

### 4.2 Retroviral and Lentiviral Particle Production and Transduction

Retroviral vectors coding B‐RAF:ER fusion protein (Woods et al. 1997) or ER:H‐RasG12V (Young et al. 2009) were used to generate MRC5/RAF:ER cells or MRC5/RAS:ER respectively, in which the oncogene can be activated by tamoxifen (4‐OHT) treatment. In order to overexpress HIF transcription factors, HA‐HIF1A P402A/P564A‐pBabe‐puro (Addgene plasmid #19005) and HA‐HIF2alpha‐P405A/P531A‐pBabe‐puro (Addgene plasmid #19006) plasmids were used (Yan et al. 2007). In order to overexpress ANGPTL4, ANGPTL4‐pBabe‐puro (Addgene plasmid #19156) plasmid was used (Padua et al. 2008). For control, empty vector pBabe‐puro (Addgene plasmid #1764) was used (Morgenstern and Land 1990).

Lentiviral vectors coding shHIF2A, pLV[shRNA]‐Hygro‐U6 > hEPAS1[shRNA#1 targeted sequence GCGCAAATGTACCCAATGATA] (VectorBuilder) was used. IL1A overexpression was performed using pLV[Exp]‐Hygro‐CMVhIL1A[NM_000575.5] (VectorBuilder).

To produce retroviral or lentiviral particles, 293GP and 293T cells were transfected using the PEIpro transfection reagent (Ozyme) according to the manufacturer's recommendations. Two days after transfection, the viral supernatant was mixed with fresh medium and hexadimethrine bromide (8 μg/mL; Sigma Aldrich). The viral dilution for retrovirus was 1/4, while for lentivirus it was 1/60. Then MRC5 cells with viral supernatant were centrifuged for 30 min at 2000 RPM and incubated for 6 h.

### 4.3 siRNA Transfection

MRC5 cells were reverse transfected with 15 nM ON‐TARGET plus SMART pool of siRNAs targeting RELA (siRELA), CEBPB (siCEBPB), ANGPTL4 (siANGPTL4), FURIN (siFURIN), PCSK5 (siPCSK5). siRNAs were incubated for 15 min with Dharmafect 1 Transfection Reagent (Horizon Discovery) at 0,6% in DMEM medium without serum or antibiotics. The transfection was carried out overnight. The next day cells were infected with virus particles or treated with 4‐OHT, as referenced previously.

### 4.4 RNA Extraction

RNA extraction was done using the “NucleoZOL” kit (Macherey‐Nagel) following the manufacturer's protocol. The concentration of RNA was measured by spectrophotometry, using “NanoDrop” (Thermo Fisher Scientific).

### 4.5 Neutrophil Preparation and Ex Vivo Activation Assays

Blood from healthy donors was collected via the Etablissement Français du Sang and withdrawn in EDTA or Heparin‐coated vacutainers. Red cells from heparinized whole blood were lysed with PharmLyse 1× solution (BD), and leukocytes were incubated with 1:8 diluted filtered culture supernatants (from siCTRL‐, siANGPTL4‐ or siRELA‐transfected, senescent or not, MRC5) for 15 min at 37°C—5% CO_2_. Phosphorylation of STAT5, using pSTAT5 antibody (STAT5(PY694), BD), was evaluated using a panel of antibodies to identify neutrophils [CD45+ (HI30, BD), CD66b + (G10F5, BD), CD15high (HI98, BD), CD14‐ (M5E2, Biolegend), Siglec8‐ (REA1045, Miltenyi)]. For CD63 staining, circulating neutrophils were obtained from EDTA‐whole blood by negative immune‐selection using the EasySEP Direct Human Neutrophil Isolation Kit (Stemcell) following the manufacturer's instructions. Isolated neutrophils were treated with 1:10 diluted filtered culture supernatants for 10 h at 37°C—5% CO_2_ and CD63 was stained using anti‐human CD63 (H5C6, BD). pSTAT5 and CD63 stainings were evaluated by multiparametric flow cytometry (LSR Fortessa—BD Biosciences) and analyzed using the FlowJo 10.4 software (Tree Star Inc).

### 4.6 Reverse Transcription (RT) and Real Time Quantitative Polymerase Chain Reaction (qPCR)

RT was performed starting from 800 ng of total RNA by using “Dynamo cDNA synthesis” kit (Thermo Fisher Scientific), following the protocol provided by the producer. In the end of the protocol cDNA was diluted 5× by adding RNase‐free water to perform qPCR with ONEGreen Fast qPCR Premix (Ozyme) and Thermocycler FX96 (BioRad). The list of primers used is indicated in Table S1. The mRNA levels were calculated using the *C*_t_ (ΔΔ*C*_T_) method; genes were normalized to GAPDH.

### 4.7 Western Blot

Whole cell extract proteins were purified by adding 2× Laemmli buffer. Filtered (except if specified otherwise in the figure legend) supernatant proteins were obtained from 500 μL of cell culture with white DMEM medium (Life Technologies) concentrated 10 times using Amicon Ultra 3 K columns (Millipore). Then, proteins were separated by SDS‐PAGE in Tris–HCl–Glycine–SDS TGS pH 8.5 (Euromedex). Following separation, proteins were transferred onto a nitrocellulose membrane (BioRad) using Tris‐Glycine pH 8.5 Buffer (Euromedex). The membranes were then blocked for 1 h with 5% milk in TBS‐T 0.05% and subsequently incubated overnight at 4°C with primary antibodies in TBS‐T with 5% milk: α‐ANGPTL4 (sc‐373761, Santa Cruz, 1/250), α‐IL6 (sc‐28343, Santa Cruz, 1/500), α‐FURIN (18413‐1‐AP, Proteintech, 1/1000), α‐TUBULIN (T6199‐100, Sigma, 1/5000). α‐HIF1A (610958, BD Biosciences, 1/1000), HIF2A (NB100‐132, Novus Biologicals, 1/1000), INHBA (GTX108405, GeneTex 1/1000). Membranes were then incubated with mouse anti‐human HRP‐conjugated IgG4 secondary antibody (Life science) for 1 h at room temperature in TBS‐T with 5% milk. Signal detection was performed using Pierce ECL Western blotting substrate (Life Technologies) or Clarity Max ECL substrate kit (Bio‐Rad) using the ChemiDoc imaging system (Bio‐Rad Laboratories).

### 4.8 Senescence‐Associated β‐Galactosidase Analysis and Crystal Violet

In vitro, cells were washed with PBS, fixed in 2% formaldehyde/0.2% glutaraldehyde for 5 min. Cells were then rinsed twice in PBS 1X, and incubated at 37°C overnight in SA‐β‐galactosidase staining solution as previously described (Debacq‐Chainiaux et al. 2009). In vivo, cryosections of the lungs were prepared and the experiments performed as previously described (Debacq‐Chainiaux et al. 2009).

For crystal violet staining, cells were washed with PBS, fixed in 3.7% formaldehyde for 10 min and stained with crystal violet solution.

### 4.9 ELISA

Quantification of ANGPTL4 and IL6 proteins in filtered and concentrated cell culture supernatants was performed using respectively “Human Angiopoietin like4 ELISA kit” (Thermo Scientific), ELISA for Human IL‐8/CXCL8 DuoSet (Bio‐Techne), ELISA for Human IL‐1 alpha/IL‐1F1 DuoSet (Bio‐Techne), and ELISA for Human IL‐6 DuoSet (R&D Systems) according to the manufacturer's instructions.

### 4.10 Intranasal Infection and Lesion Quantification

LSL‐KrasG12D (Jackson et al. 2001) and R26R‐LSL‐tdTomato mice which have been previously described were crossed to generate males and females double heterozygote KrasG12D,R26RTomato animals (termed KT). All experiments were performed in accordance with the regulations for animals used for scientific purposes governed by the European Directive 2010/63/EU. Protocols were validated by the Animal Ethics Evaluation Committee number C2EA15 and authorized by the French Minister of Research and Innovation under the reference APAFIS#28448. Viral infections were performed using an adapted intranasal instillation described in (Santry et al. 2017). Briefly, isoflurane‐anesthetized mice were restrained by the scruff at a 45° angle, the mouth was gently pinched to keep it shut and to prevent mouth breathing. Then, 40 μL of the Lenti‐CRE virus (10^6^ TU), produced and tittered by Vector Builder, was delivered using a pipette and 100 μL tip, drop wise over the nares. After virus delivery, the mouse was maintained in this position for approximately 30 s to allow passive distribution of the viral particles into the lower respiratory tract. Fifteen weeks post‐infection, lungs were collected after cervical dislocation and fixed in formalin for 24 h. Tissue samples were then stored in 70% EtOH until paraffin embedding and processing. In order to quantify neoplastic lesions, six sections spaced by 50 μm were collected. The slides were then stained with Hematoxylin–eosin and scanned on the Antipath CRCL core facility. The sections were visualized using QuPath software.

### 4.11 Immunohistochemistry

Paraffin tissue slides were deparaffinized using Xylene (Sigma Aldrich) and EtOH (Sigma Aldrich). Inhibition of peroxidases was then performed using a hydrogen peroxidase solution (Sigma Aldrich) 3% in H_2_O for 30 min at RT. After the slides were washed 3 times, Heat‐induced Epitope Retrieval was performed by microwaving in Antigen Unmasking Solution (citric acid‐based, pH = 6.0) (H‐3300, Vector Laboratories) for 5 min at 800 W, then for 10 min at 450 W. After cooling, the slides were washed 3 times with PBS and blocking was performed with antibody diluent reagent (#003118, Invitrogen) for 1 h at RT. Primary antibodies were diluted in diluent reagent and incubation was performed at 4°C, overnight. The following primary antibodies were used: ANGPTL4 (NBP2‐19016, Novus); tomato (AB0040‐200, Origene); HIF2A (Lau et al. 2007), p21 (M7202, Dako); γH2AX (2577S, Cell Signaling); CCL3 (2190–1, Proteintech); FURIN (18413–1‐AP Proteintech); and IL1A—Goat Polyclonal mouse‐IL1A (AF‐400‐NA, R&D systems). Antibodies were validated according to their validation/citation in the literature (using the citeab website), validation by the providers (technical sheets), and/or expected staining profiles (pattern and/or intensity).

Slides were then washed 3 times with PBS, and incubation with secondary antibodies: Horse Anti‐Rabbit IgG Antibody (BA‐1100, Vector Laboratories), Horse Anti‐Goat IgG Antibody (BA‐9500, Vector Laboratories) diluted 1/200 was performed at RT for 45 min. After 3 washes with PBS, signal amplification was performed using the VECTASTAIN Elite ABC kit (PK‐6100, Vector Laboratories) for 45 min at RT. The signal was detected using the diaminobenzidine tetrahydrochloride substrate kit (SK‐4100, Vector Laboratories). Then hematoxylin (Sigma Aldrich) was used as counterstaining and slides were dehydrated using EtOH (Sigma Aldrich) and xylene and mounted with Pertex (Histolab).

### 4.12 Gene Set Enrichment Analysis (GSEA)

GSEA was performed using the GSEA website (www.broadinstitute.org/gsea/). Briefly, Pre‐ranked GSEA was conducted on the ranked list of relative gene expression either between senescent versus control conditions using transcriptome data for Figure 2 or between lung tumors expressing High ANGPTL4 mRNA (upper quartile) versus lung tumors expressing low ANGPTL4 mRNA (lower quartile) using LUAD TCGA data for Figure 5. Graphs were generated from the GSEA website.

### 4.13 Cut & Tag

Genome‐wide localization of HIF2A was performed by Cut & Tag as described (Kaya‐Okur et al. 2020) using 100,000 MRC5 cells ectopically expressing HIF2A from the pBabe vector, or control MRC5 cells containing only the pBabe vector. Two biological replicates were done for the MRC5‐HIF2A cells. HIF2A was mapped with the mouse monoclonal anti‐HIF2A antibody Ep190b (1 μg of antibody per Cut & Tag reaction). We also performed H3‐K27me3 Cut & Tag reactions in parallel as a positive control using an Abcam mouse monoclonal anti‐H3‐K27me3 (ab6002). We used rabbit anti‐mouse‐IgG (Millipore AP160) as the secondary amplifying antibody. The proteinA‐Tn5 fusion protein was prepared as described (https://www.protocols.io/view/3xflag‐patn5‐protein‐purification‐and‐meds‐loading‐j8nlke4e5l5r/v1) and used for the Cut & Tag reactions at a dilution of 1/100. Libraries of fragmented DNA were prepared by 13 cycles of PCR amplification for H3‐K27me3 and 15 cycles of amplification for HIF2A using Nextera primers with Illumina index sequences. Primers were removed by purification of DNA greater than 100 bp using AMPure beads. The DNA libraries were quantified by Qubit and by Tape Station D1000 analysis. Libraries were sequenced on an Illumina NextSeq 500 sequencer with paired‐end 42 nucleotide sequencing cycles. Sequences were demultiplexed with bcl2fastq2 and adapters were trimmed with Cutadapt. The quality of the reads was controlled with FastQC. The paired‐end reads were mapped to the human genome (hg19) using the parameters suggested by the Henikoff lab (https://yezhengstat.github.io/CUTTag_tutorial/). The resulting bam files were sorted, indexed, and duplicate reads were removed with samtools. Macs2 callpeak in BAMPE mode was used to identify 791 differential peaks in MRC5‐HIF2A versus the MRC5‐pBabe control. Peaks were annotated with Homer annotatePeaks (Table S2).

### 4.14 Chromatin Immunoprecipitation (ChIP)‐qPCR Assay

ChIP was performed using the SimpleChIP Enzymatic Chromatin IP Kit (Cell Signaling Technology, #9003). Briefly, 4‐OHT‐induced senescent or non‐senescent MRC5/RAF:ER cells were fixed in 1% formalin for 10 min. The nucleus fraction was isolated according to the manufacturer's protocol. Chromatin was digested with micrococcal nuclease, and the nuclei were subsequently disrupted by sonication. The resulting chromatin was immunoprecipitated using 5 μg anti‐HIF‐2α antibody (NOVUS, NB100‐122) or normal rabbit IgG (CST, #2729P). DNA was purified and analyzed by quantitative PCR. Primers targeting the ANGPTL4 promoter region were designed as below. ANGPTL4 set1; Fwd 5′‐CAC TGG GGT TTT CCT CCC TC‐3′, Rev. 5′‐CAG GCT GCC ACT CAT ACA CT‐3′. ANGPTL4 set2; Fwd 5′‐ATT TCA CAC TAG AGG CGG GC‐3′, Rev. 5′‐CAG GCC TTC CTC TAC GAA CC‐3′. Actin promoter; Fwd 5′‐AGC GCG GCT ACA GCT TCA −3′, Rev. 5′‐CGT AGC ACA GCT TCT CCT TAA TGT‐3′.

### 4.15 Statistical Analysis

Statistical analysis was performed and graphs were created using Graph Pad Prism 10. The number of independent biological replicates is indicated in the figure legends (*n* ≥ 3 unless specified otherwise). No points were excluded for the analysis, unless indicated otherwise in the figure legends. Statistical tests are indicated in the figure legends. For RT‐qPCR, statistical analysis was performed on GAPDH normalized ΔCT values between conditions.

## Author Contributions

G.M., A.H., A.M., H.S.‐Z., T.M., A.P., L.M., P.L., J.G., J.‐M.F. performed in vitro experiments. G.M., D.G., J.‐J.M., M.C. performed in vivo experiments. G.M., M.‐C.M., J.G., S.A., P.D., H.K., C.M., N.S.T., P.B., J.‐M.F., D.B. designed the experiments and the results were analyzed by all the co‐authors. J.‐M.F. and D.B. designed the overall study and co‐supervised the work. G.M., J.‐M.F. and D.B. wrote the manuscript with input from all authors.

## Funding

This work was supported by the Ligue Régionale contre le Cancer (Comités de Savoie et du Puy‐de‐Dôme) to J.‐M.F., by Fondation ARC contre le Cancer (ARCPJA2021060003918) to D.B. and by INCA Institut National du Cancer (INCa_14908; INCA_16694) to D.B. C.M. was supported by INCA PLBio_2020. G.M. (FDT202304016783) and H.S.‐Z. (SPF202409019572) were supported by the Fondation pour la Recherche Médicale FRM. Institut Convergence PLAsCAN, ANR‐17‐CONV‐0002 also supports the project.

## Conflicts of Interest

The authors declare no conflicts of interest.

## Supporting information


**Supplementary Figures:** acel70307‐sup‐0001‐Figures.pdf

**Table S1:** acel70307‐sup‐0002‐TableS1.pdf

**Table S2:** acel70307‐sup‐0003‐TableS2.xlsx.

## Data Availability

The data that support the findings of this study are available from the corresponding authors. Cut & Tag sequencing data were deposited at the EMBL‐EBI ArrayExpress under accession number E‐MTAB‐13732. Transcriptome datasets were deposited in GEO under the accession number GSE310703.
