# Supplementary material for: The Proinflammatory Secretome of Senescent Cells Can Be Controlled by a HIF2A‐Dependent Upregulation and a FURIN‐Dependent Cleavage of the ANGPTL4 Secreted Factor
Source: Aging Cell. 2025 Dec 5;25(1):e70307. doi: 10.1111/acel.70307 (PMC12741039; doi:10.1111/acel.70307)
Supplement: Supplementary file 1 — Supplementary Figures: acel70307‐sup‐0001‐Figures.pdf [file ACEL-25-e70307-s001.pdf]

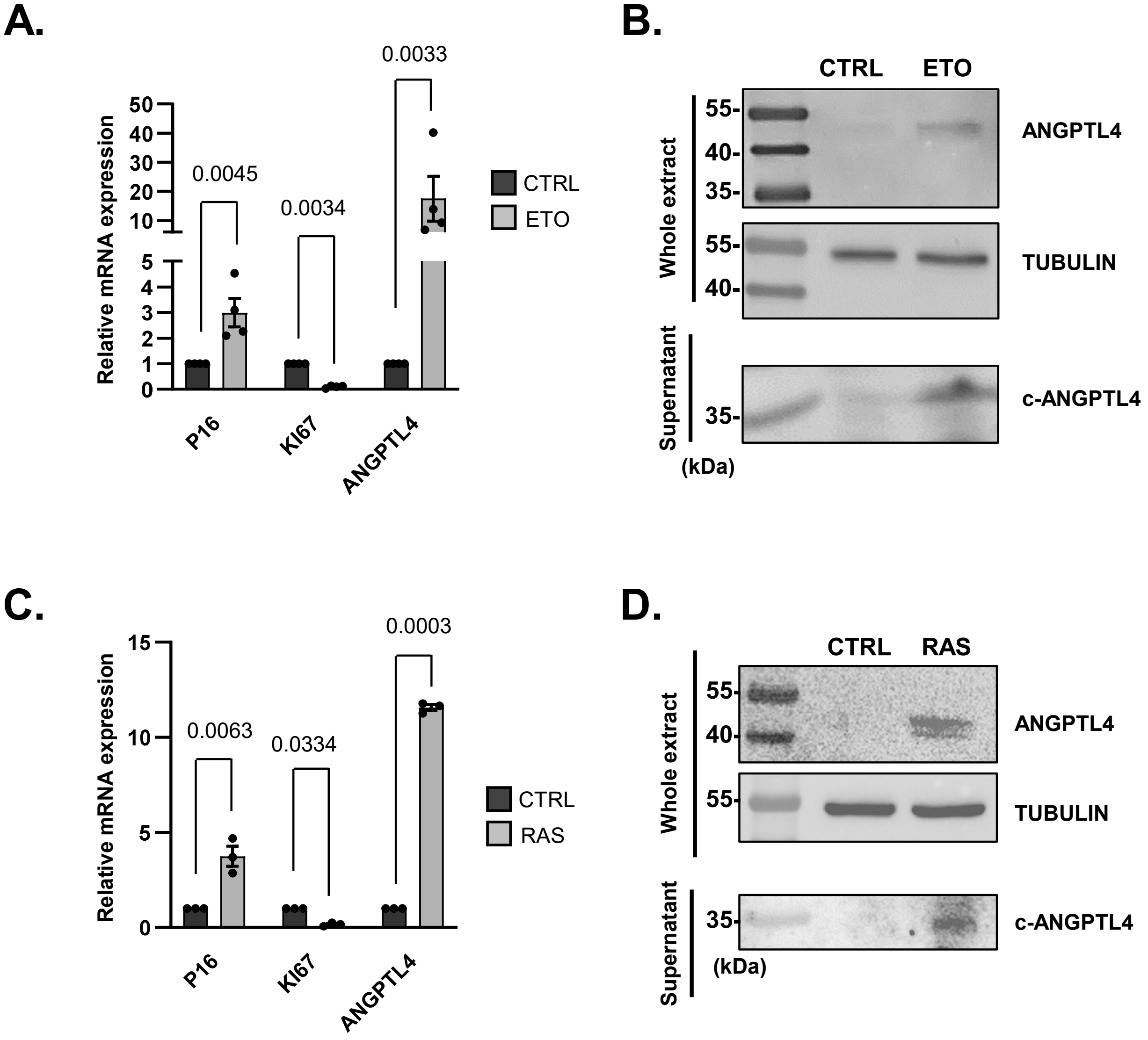

Figure S1: (A-B) MRC5 cells were not treated (CTRL) or treated with Etoposide (ETO), and **(A)** relative mRNA expression of *P16*, *KI67* and *ANGPTL4* genes by using RT-qPCR, or **(B)** western blots were performed against the indicated proteins. **(C-D)** MRC5/RAS:ER cells were not treated (CTRL) or with 4-OHT (RAS), and **(C)** relative mRNA expression of *P16*, *KI67* and *ANGPTL4* genes by using RT-qPCR, or **(D)** western blots were performed against the indicated proteins. For RT-qPCR, data are presented as mean +/- SEM of n=3 or 4 independent experiments and Paired t-tests are indicated. Blots are representative of n=3 experiments.

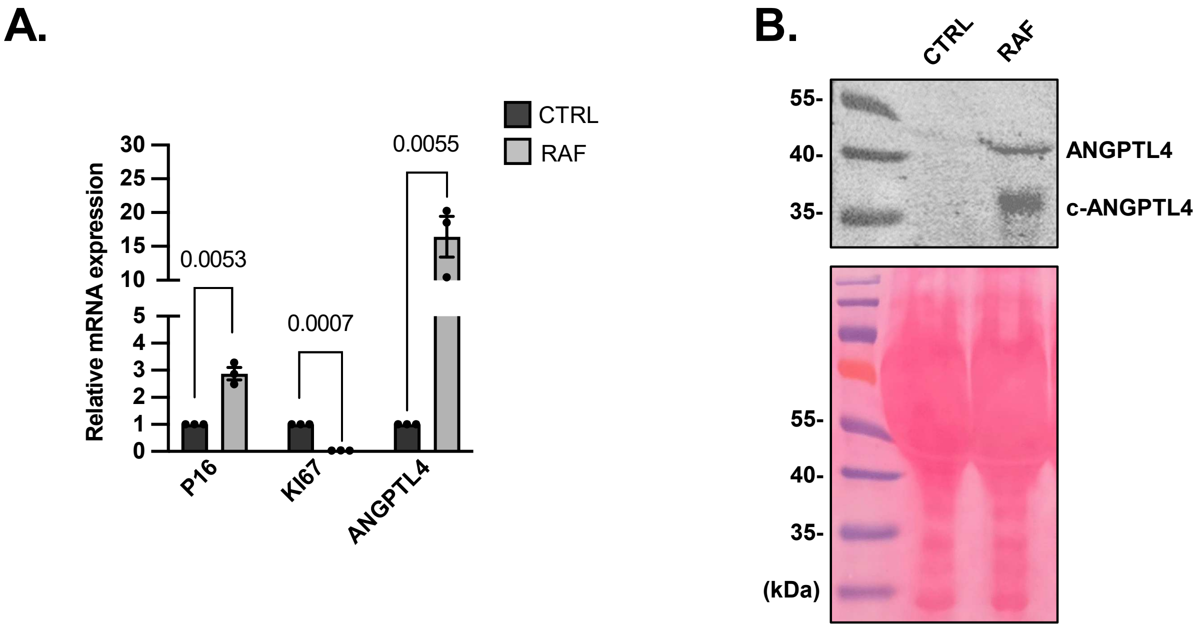

Figure S2: (A) Relative mRNA expression of *P16*, *KI67* and *ANGPTL4* genes by using RT-qPCR, in HDF/RAF:ER cells not treated (CTRL) or treated with 4-OHT (RAF). Mean +/- SEM of n=3 independent experiments. Paired t-test results are indicated. **(B)** Non Filtered supernatants were used to analyze expression of ANGPTL4 and its cleaved form (c-ANGPTL4). Representative of n=3 experiments.

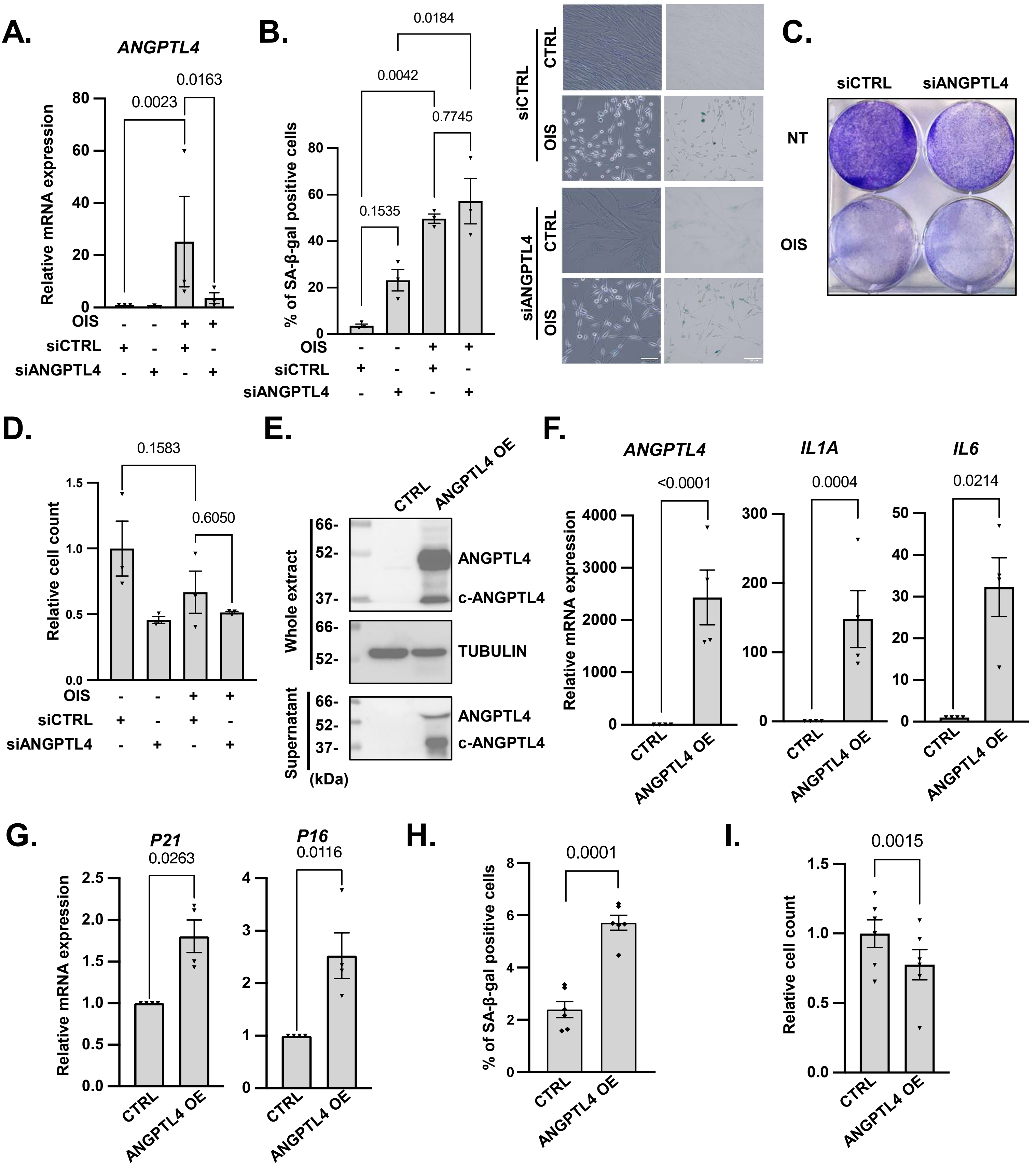

Figure S3: **(A-D)** MRC5/RAF:ER cells were transfected with control siRNA (siCTRL) or siRNA targeting ANGPTL4 (siANGPTL4) and treated (+) or not (-) with 4-OHT to induce senescence (OIS). **(A)** Relative ANGPTL4 mRNA expression measured by RT-qPCR. Mean +/- SEM of n=3 independent experiments. Paired one-way ANOVA tests are indicated. **(B)** Images and quantification of SA-β-gal positive cells. Mean +/- SEM of n=3 independent experiments. Paired one-way ANOVA tests are indicated. **(C)** Crystal violet assay. Representative of n=3 independent experiments. **(D)** Relative cell number. Mean +/- SEM of n=3 independent experiments. Paired one-way ANOVA tests are indicated. **(E-I)** MRC5 cells were infected with retroviral vectors encoding ANGPTL4 (ANGPTL4 OE) or empty vector (CTRL). **(E)** Western blot analysis of ANGPTL4 expression Full length ANGPTL4 detected and TUBULIN loading control in whole extract and c-ANGPTL4 detected in cell supernatants. Representative picture of n=3 independent experiments. **(F)** Relative mRNA expression of *ANGPTL4*, *IL1A* and *IL8* genes were analysed by RT-qPCR. Mean +/- SEM of n=4 independent experiments. Paired one-way ANOVA tests are shown. **(G)** Relative mRNA expression of *p21* and *p16* cyclin-dependent kinases inhibitors. Mean +/- SEM of n=4 independent experiments. Paired one-way ANOVA tests are shown. **(H)** quantification of SA-β-gal positive cells. Mean +/- SEM of n=5 independent experiments. Paired t-tests are indicated. **(I)** Relative cell count. Mean +/- SEM of n=6 independent experiments. Paired t-tests are indicated.

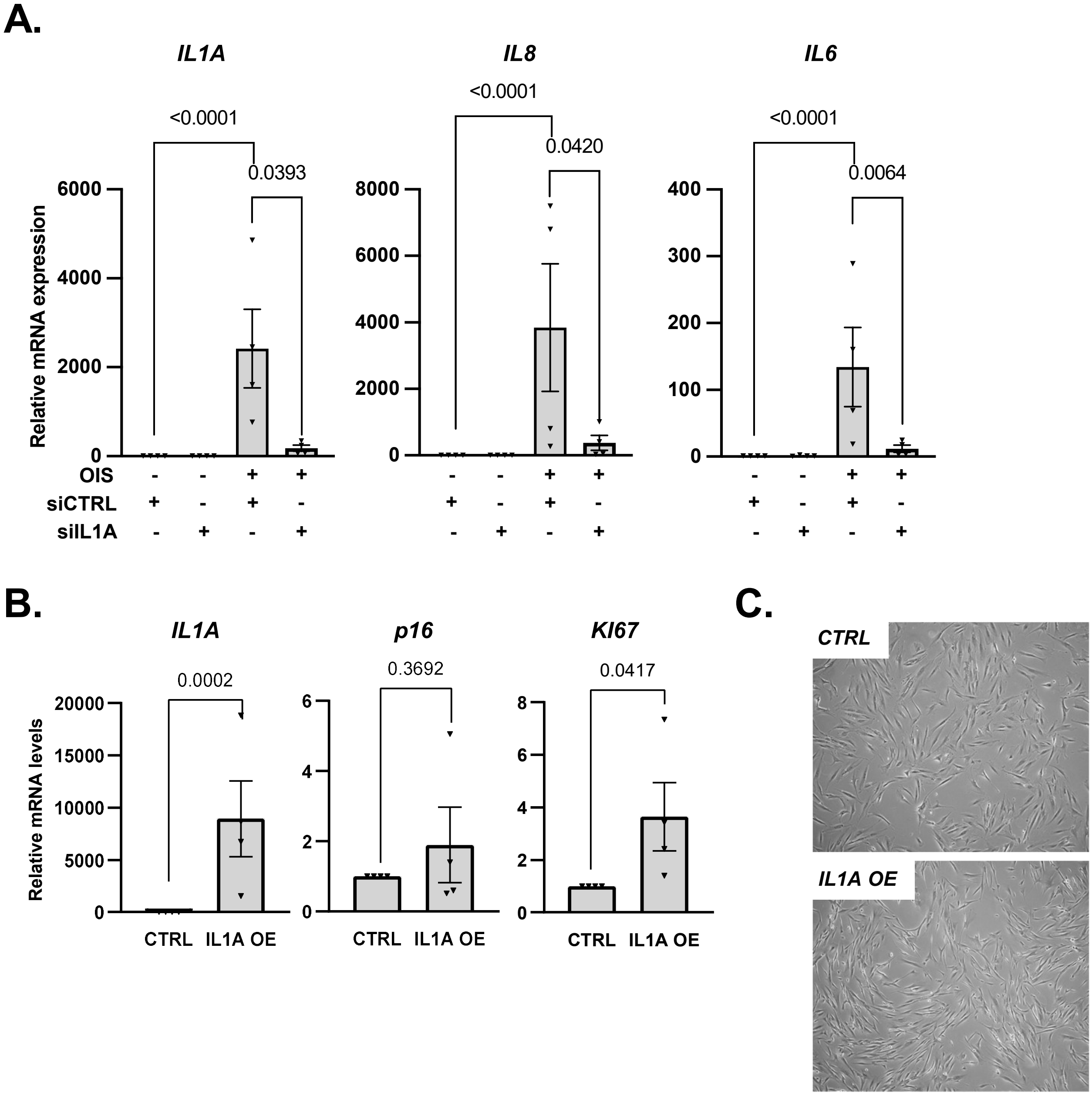

Figure S4: (A) RT-qPCR analysis of IL1A and pro-inflammatory SASP members in MRC5/RAF:ER cells treated or not with 4-OHT to induce senescence and transfected with control (siCTRL) or IL1A (siIL1A) siRNA. Mean +/- SEM of n=4 independent experiments. Paired one-way ANOVA tests are shown. **(B-C)** Three days after infection with a control (CTRL) or IL1A (IL1A OE) encoding lentiviral vector, (B) RNAs were prepared and qPCR against the indicated genes performed (Mean +/- SEM of n=3 independent experiments. Paired one-way ANOVA tests are shown), or (C) images of cells were taken to illustrate cell density.

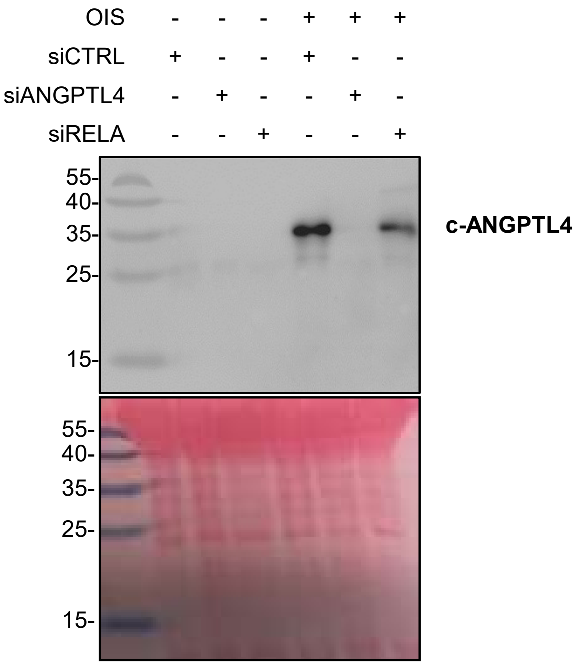

Figure S5: Western blot analysis of c-ANGPTL4 expression on supernatants of MRC5/RAF:ER transfected with the indicated siRNA and not treated (CTRL) or treated with 4-OHT to activate RAF (OIS). Representative picture of n=3 independent experiments.

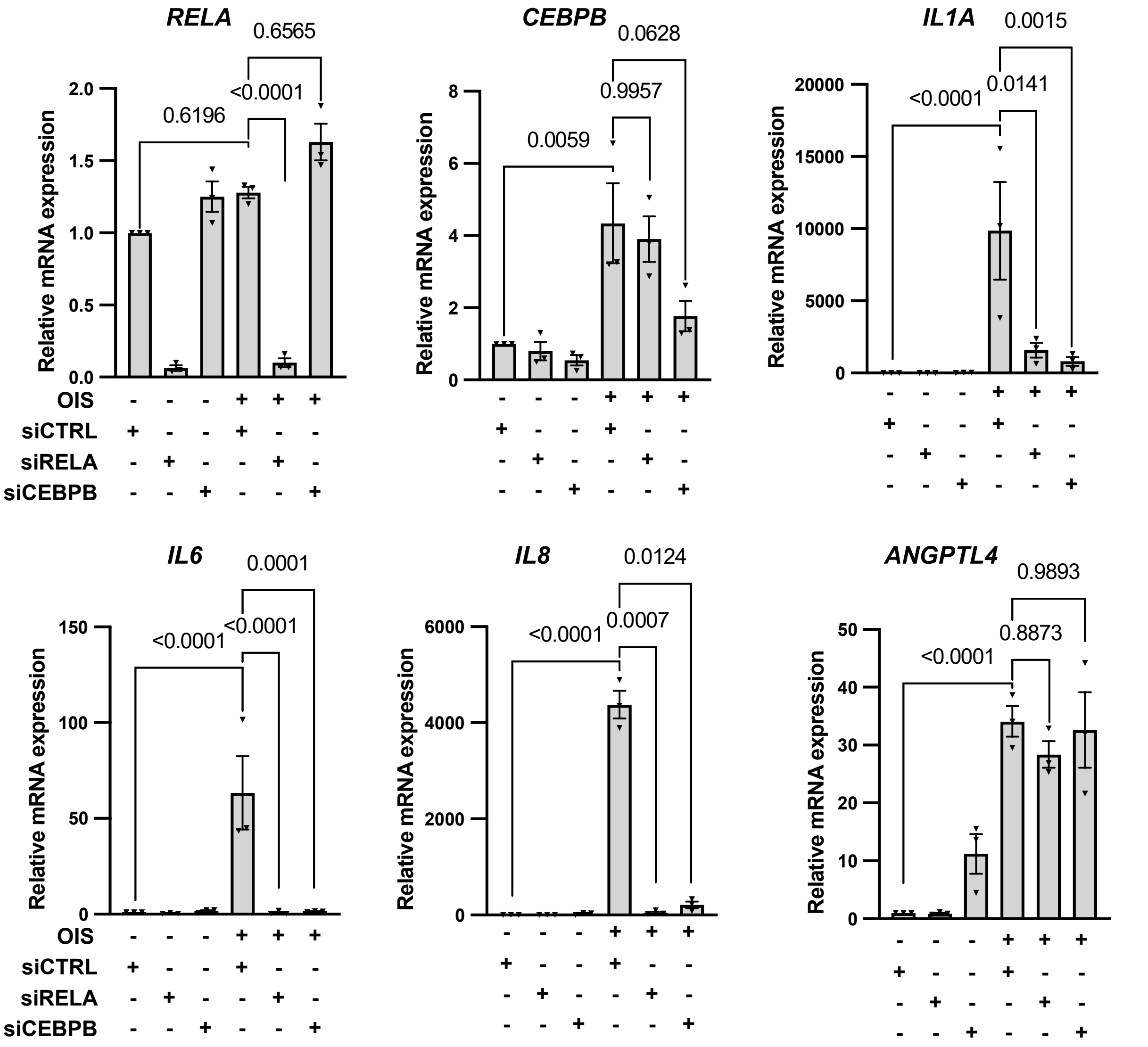

Figure S6: RT-qPCR analysis of *RELA*, *CEBPB*, *IL1A*, *IL6*, *IL8* and *ANGPTL4* gene expression in MRC5/RAF:ER cells transfected with control siRNA (siCTRL), or siRNA targeting RELA (siRELA), CEBPB (siCEBPB) and treated (+) or not (-) with 4-OHT to induce senescence (OIS). Mean +/- SEM of n=3 independent experiments. Paired one-way ANOVA on ΔCT values.

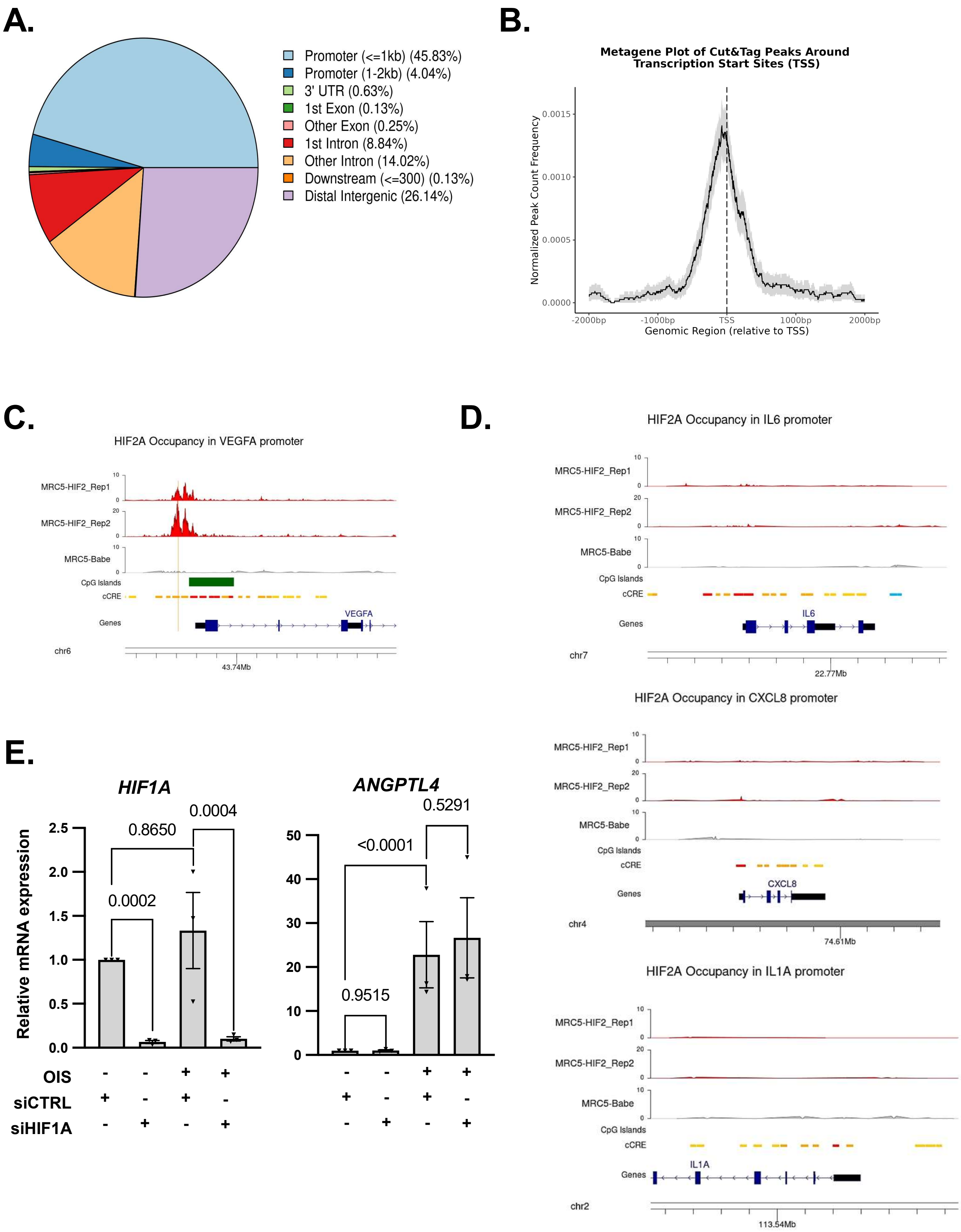

Figure S7: (A) Annotation and distribution of significant HIF2A peaks identified by Cut & Tag across genomic features. **(B)** Significant HIF2A peaks was profiled over the TSS of corresponding genes to generate a metagene profile. **(C-D)** HIF2A peaks at the VEGFA promoter (C) or at SASP gene promoters (IL6, IL8, IL1A) (D) in MRC5 cells infected with HIF2A (HIF2) or control (Babe) retroviral particles. Two independent experiments were performed (HIF2A-Rep1, HIF2A-Rep2). **(E)** RT-qPCR analysis of HIF1A and ANGPTL4 during OIS in MRC5 after transfection with the indicated siRNA. Mean +/- SEM of n=3 independent experiments. Paired one-way ANOVA tests are shown.

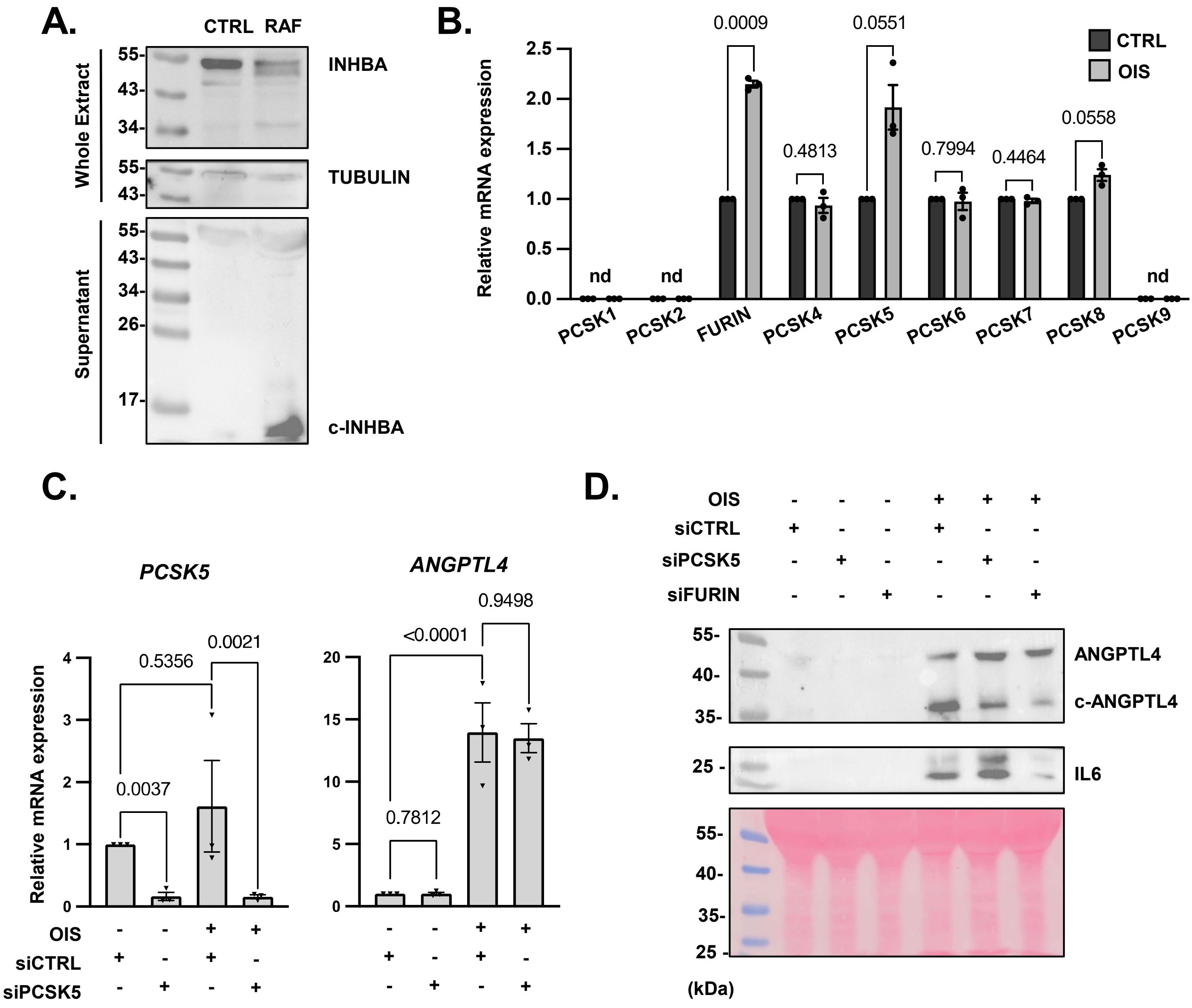

Figure S8: (A) Western blot analysis of INHBA expression in MRC5/RAF:ER not treated (CTRL) or treated with 4-OHT to activate RAF (OIS). Full length INHBA detected and TUBULIN loading control in whole extract and cleaved INHBA (c-INHBA or Activin A) detected in cell supernatants. Representative picture of n=3 independent experiments. **(B)** Expression levels of the indicated PCSK one day after RAF activation (GSE310703). nd mean not detected. **(C)** RT-qPCR analysis against the indicated genes in MRC5/RAF:ER not treated (CTRL) or treated with 4-OHT (RAF) after transfection by the indicated siRNA. **(D)** Western blot analysis of ANGPTL4 and IL6 in non filtered supernatants. Representative picture of n=3 independent experiments.

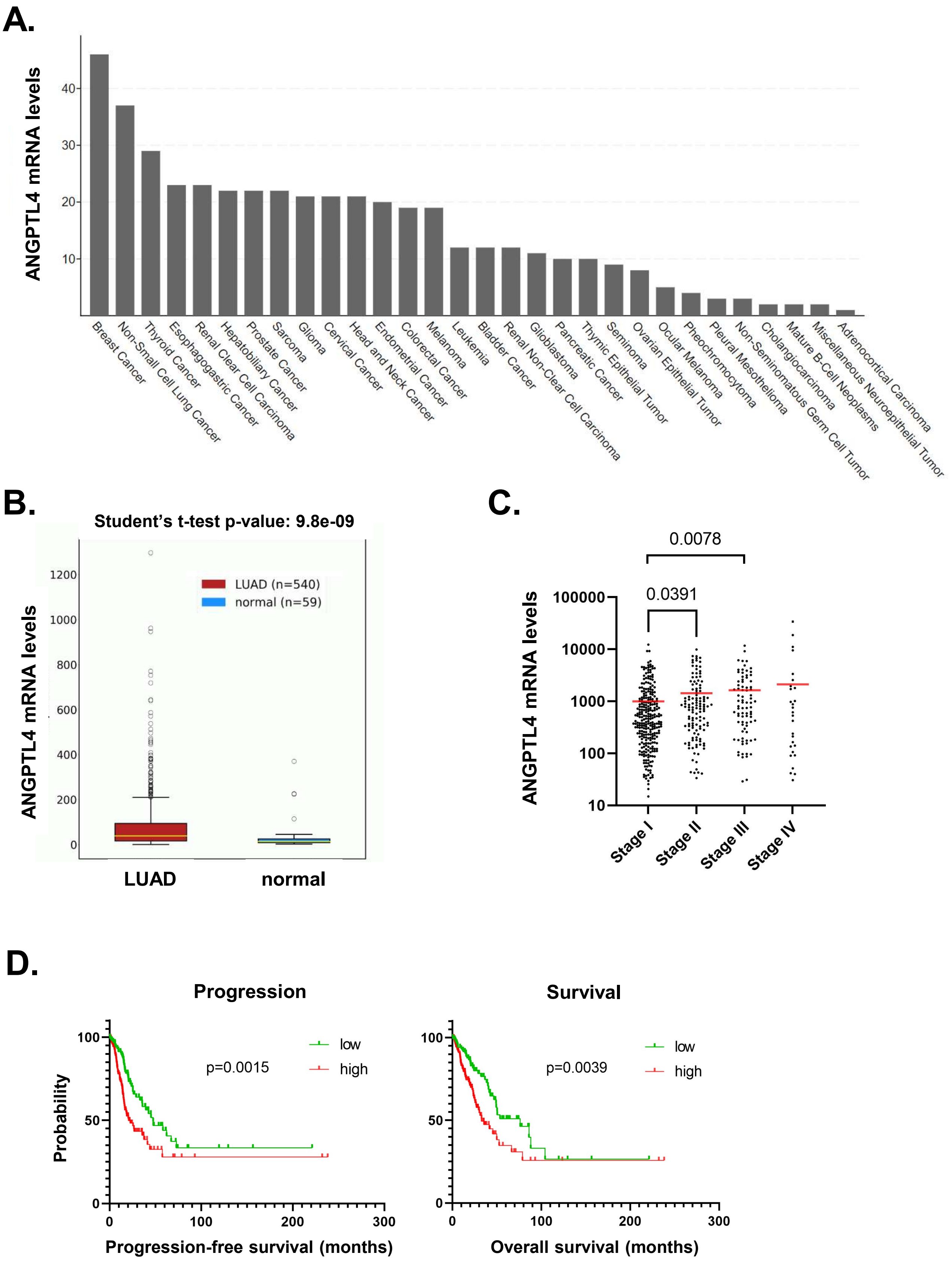

Figure S9: ANGPTL4 is highly expressed and associated to poor prognosis lung tumors. (A) ANGPTL4 mRNA expression in different cancer types according to TCGA database using cBioPortal website. **(B)** ANGPTL4 mRNA expression in lung adenocarcinoma (LUAD) compared to normal tissue. These data were extracted from https://oncodb.org/. **(C-D)** Clinico-biological data were extracted LUAD TCGA datasets from cBioPortal and analysed. Neoplasm Disease Stage American Joint Committee on Cancer Code was used to distinguish between tumor stages. Individual values and the mean are shown. Statistical significance determined by Kruskal-Wallis tests and Dunn multiple comparisons. **(C)** ANGPTL4 mRNA expression comparison according to LUAD grades. **(D)** Kaplan-Meier curves for LUAD progression and patient. survival depending of ANGPTL4 expression. Log-rank (Mantel-Cox) tests were used to determine statistical significance.

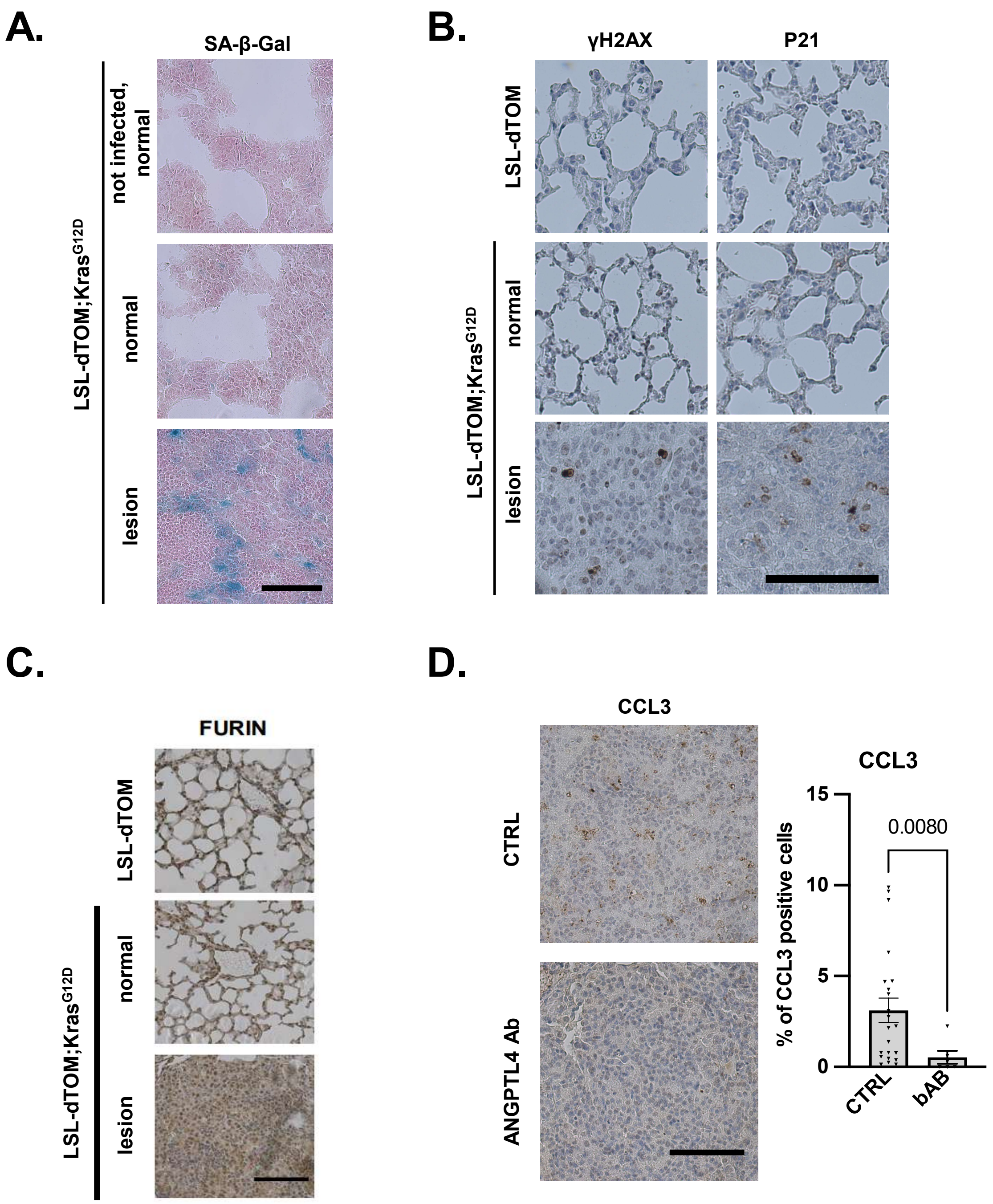

Figure S10: (A-C) Lungs were prepared 15-20 weeks after CRE-encoding lentivirus infection. **(A)** Representative images of SA-b-Gal assays performed on frozen lungs. Scale bar: 100µm. **(B-C)** Immunohistochemistry were performed against γH2AX and p21 **(B)** or FURIN **(C)** and representative images are shown. Scale bar: 100µm. **(D)** LSL-dTOM;Kras^{G12D} mice were infected with a CRE-encoding lentivirus. 5 weeks post-infection they were treated with ANGPTL4 blocking antibody (bAb) for 10 weeks. Immunohistochemistry analysis of CCl3 staining in mouse neoplastic lung lesions treated with ANGPTL4 bAb. (from N=6 mice per group). Upper panel: representative picture of the staining, scale bar: 100µm. right panel: quantification result, percentage of positive cells per lesion. (bAb), n=22 lesions; and not treated (CTRL), n=6 lesions after removing outliers using GraphPad software. Mean +/- SEM, Mann Whitney tests.
