# Supplementary material for: The Proinflammatory Secretome of Senescent Cells Can Be Controlled by a HIF2A‐Dependent Upregulation and a FURIN‐Dependent Cleavage of the ANGPTL4 Secreted Factor
Source: Aging Cell. 2025 Dec 5;25(1):e70307. doi: 10.1111/acel.70307 (PMC12741039; doi:10.1111/acel.70307)
Supplement: Supplementary file 2 — Table S1: acel70307‐sup‐0002‐TableS1.pdf [file ACEL-25-e70307-s002.pdf]

Genes	Forward	Revers
KI67	TCAAGGAACTGATTCAGGAGAAG	GTGCACTGAAGAACACATTTCC
P21	TCACTGTCTTGTACCCTTGTGC	GGCGTTTGGAGTGGTAGAAA
P16	CGGTCGGAGGCCGATCCAG	GCGCCGTGGAGCAGCAGCAGCT
ANGPTL4	GACAAGAACTGCGCCAAGA	GCCGTTGAGGTTGGAATG
RELA	TCATGAAGAAGAGTCCTTTCAGC	CTGGCTTGGGGACAGAAG
CEBPB	CGCTTACCTCGGCTACCA	ACGAGGAGGACGTGGAGAG
IL6	GATGAGTACAAAAGTCCTGATCCA	CTGCAGCCACTGGTTCTGT
IL8	AGACAGCAGAGCACACAAGC	ATGGTTCCTTCCGGTGGT
IL1A	GGTTGAGTTTAAGCCAATCCA	TGCTGACCTAGGCTTGATGA
FURIN	GTGTGGTGTGCGAGGAA	CTCGGTGCTATAGTGCGTATC
PCSK5-Var2	CTGCCCTAGTGGGTATCTCTTA	CAAGCATACAGAAGCCTCCTT
SPP1	GAGGGCTTGGTTGTCAGC	CAATTCTCATGGTAGTGAGTTTTCC
HIF2A	GACATGAAGTTCACCTACTGTGATG	GCGCATGGTAGAATTCATAGG
HIF1A	TTTTTCAAGCAGTAGGAATTGGA	GTGATGTAGTAGCTGCATGATCG
GAPDH	AGCCACATCGCTCAGACAC	GCCCAATACGACCAAATCC

Table S1: List of qPCR primers to assess mRNA levels.
